# The Effectiveness of Nonpharmacological Interventions in the Management of Chemotherapy Physical Side Effects: A Systematic Review

**DOI:** 10.3390/healthcare12181880

**Published:** 2024-09-19

**Authors:** Valentina Elisabetta Di Mattei, Gaia Perego, Francesca Milano, Francesca Gatti

**Affiliations:** 1 School of Psychology, Vita-Salute San Raffaele University, 20132 Milan, Italy; dimattei.valentina@hsr.it (V.E.D.M.); perego.gaia@hsr.it (G.P.); francescagatti.mail@gmail.com (F.G.); 2 Clinical and Health Psychology Unit, IRCCS San Raffaele Scientific Institute, 20132 Milan, Italy; 3 Department of Psychology, University of Milano-Bicocca, 20132 Milan, Italy

**Keywords:** chemotherapy, cancer, cancer treatment, side effects, nonpharmacological interventions, integrative medicine, systematic review

## Abstract

Background: Despite advancements in cancer treatment, chemotherapy side effects significantly impact patients both physically and emotionally. While pharmacological treatments can mitigate these side effects, they may trigger additional side effects, exacerbating the overall discomfort experienced by patients; moreover, psychological factors influencing physical symptoms are beyond the reach of pharmacological interventions. Nonpharmacological interventions, however, offer the potential for complementary or alternative solutions. Objectives: This review aims to offer a comprehensive analysis of the literature on the effectiveness of nonpharmacological interventions in managing the physical side effects of chemotherapy. Methods: This review, based on a search of PubMed, PsycINFO, and Web of Science databases, identified 46 relevant studies. It categorizes interventions and evaluates their effectiveness in managing common chemotherapy side effects (fatigue, nausea, pain, diarrhea, and constipation). Results: Guided imagery, tailored exercises, and Qigong show promise in reducing fatigue, while interventions like yoga and cognitive-behavioral approaches address nausea and vomiting. Pain benefits result from guided imagery and educational interventions. Limited evidence exists for diarrhea and constipation interventions, necessitating further research. Conclusions: This review offers provisional conclusions, emphasizing the potential of integrating evidence-based nonpharmacological approaches alongside pharmacological interventions to enhance patient outcomes and reduce chemotherapy-induced side effects, considering factors such as accessibility, safety, customization, and adaptability in clinical settings.

## 1. Introduction

Cancer is a pathology constantly growing worldwide, presenting a formidable challenge to public health systems and medical communities. The 2022 report from the International Agency for Research on Cancer (IARC) [1] noted 20 million new cancer cases and 9.7 million deaths worldwide. The top 10 cancers account for over 60% of both incidence and mortality, with lung cancer being the most prevalent (12.4% of cases) and the most lethal (18.7%). In women, breast cancer is the leading cause of cancer-related deaths, while in men, lung cancer remains the most prevalent and deadliest form [2].

Cancer survival rates have shown significant improvement as of 2024, though progress has slowed in recent years. In the United States, the overall five-year relative survival rate for all cancers combined has reached approximately 67%, and ongoing therapeutic advancements are reshaping the perception of cancer as an incurable disease [3]. Advancements in treatment and management strategies are making cancer increasingly recognized as a chronic condition, allowing for prolonged control using various therapeutic modalities [4]. However, despite improved cure rates, treatments such as chemotherapy still cause significant side effects associated with nonselective action against actively proliferating normal cells [5,6].

According to the literature, between 86% and 97.4% of patients undergoing chemotherapy report experiencing at least one adverse effect, commencing as early as the initial cycle of chemotherapy and irrespective of cancer type [7,8].

Fatigue emerges as a prevalent and often debilitating side effect, garnering substantial attention within the medical community. Cancer treatment-related fatigue is defined as a subjective state of physical, emotional, and/or cognitive tiredness or exhaustion related to cancer treatment that is disproportionate to recent activity [9]. Patient-reported rates of fatigue range between 74% and 85%, underlining its significant impact on patients undergoing chemotherapy [7,10]. Furthermore, nausea and vomiting stand out as common challenges encountered by patients, with a notable 79.3% of them experiencing these adverse effects during the initial cycle of chemotherapy [10]. Moreover, other chemotherapy-induced side effects include pain, diarrhea, and constipation, which are suffered by a considerable number of patients, with rates surpassing fifty percent [7,10]. The side effects and long-term sequelae of cancer chemotherapy remain a significant concern.

Existing pharmacological treatments for chemotherapy-related side effects can prove ineffective and may even trigger additional side effects, exacerbating the overall discomfort experienced by patients, while nonpharmacological interventions are generally well-tolerated [11,12]. Furthermore, the costs associated with pharmacological interventions, in terms of financial expenses and potential adverse effects, highlight the need for alternatives [13,14]. Nonpharmacological interventions may significantly contribute to alleviating the chemotherapy side effects, thereby potentially decreasing the necessity for further expensive medications aimed at managing these side effects. An example of this is the implementation of physical exercise, which has been demonstrated to be effective while simultaneously serving as a cost-efficient alternative [13]. 

Furthermore, it is known that a multidisciplinary approach and the implementation of complementary therapies involving various professionals globally enhance the quality of life of cancer patients [15,16]. 

Despite the promising potential of nonpharmacological interventions, there remains a scarcity of studies in the literature mainly due to the difficulty in conducting studies on these treatments. Indeed, when evaluating nonpharmacological interventions, the outcomes of interest often involve subjective perceptions and experiences, such as pain relief or fatigue management, which are more difficult to measure accurately. In contrast, pharmacological studies typically focus on biomarkers, which by definition are objective [17]. These types of measurement make it easier to assess the efficacy of pharmacological treatments and standardize results across studies. Furthermore, nonpharmacological treatments are a relatively recent proposal in the field of oncology supportive care. The literature on these interventions has not yet been fully systematized, leading to gaps in our understanding and a lack of standardized protocols.

Other reviews have attempted to fill this gap, but they have focused exclusively on a singular category of nonpharmacological intervention [18] or side effects, e.g., [19,20].

The present review emphasizes the importance of managing chemotherapy side effects, especially in the context of increased life expectancy for cancer patients [21]. This review aims to address a gap in current knowledge by providing a comprehensive and updated analysis of nonpharmacological interventions for the most prevalent physical side effects of chemotherapy. It offers a novel summary of the latest research on these treatment options. By considering recent advancements and ongoing therapies, it provides a holistic assessment of the current evidence, highlighting innovative approaches and their potential benefits.

## 2. Materials and Methods

PRISMA Guidelines. The study adhered to the Preferred Reporting Items for Systematic Reviews and Meta-Analyses (PRISMA) 2020 guidelines, offering detailed recommendations for systematic review conduct. Initially issued in 2009, the PRISMA Statement underwent revision to PRISMA 2020 to accommodate changes over time [22]. This checklist, encompassing all crucial aspects, was utilized to ensure comprehensive planning and execution of the systematic review. 

Search Strategy. Potentially eligible articles were systematically searched on PubMed, PsycINFO, and Web of Science databases on 9 September 2024, using the following combinations of keywords: “psycho*” AND “chemotherapy OR chemo” AND “side effects OR adverse effects”.

We limited the search to papers published from 2002 to September 2024 to cover a sufficiently large period and ensure findings related to the current practice regarding managing chemotherapy side effects. Additionally, whenever feasible, we incorporated two search filters into the databases. These filters encompassed publications written in English and exclusively focused on adult subjects. Specifically, Web of Science did not include the subject filter, and as a result, the articles were manually chosen from this database. 

Inclusion and Exclusion Criteria. We set the following inclusion criteria:

- Studies had to report on primary research;
- Studies had to be published in a scientific journal (e.g., no dissertations or books);
- Studies had to be written in English;
- Studies had to include only adult subjects;
- Studies had to include subjects currently being treated for cancer with chemotherapy;
- Studies had to address the nonpharmacological management of physical side effects of chemotherapy involving patients.

We set the following exclusion criteria.

According to the methodology of the study, the following papers were excluded:

- Studies that proposed theories, models, guidelines, or protocols but did not assess the efficacy of an intervention;
- Studies on an intervention’s usability;
- Reviews and meta-analyses;
- Qualitative studies;
- Pilot studies, preliminary studies, and single-case studies;
- Studies regarding focus groups.

Regarding the side effects considered, the following papers were excluded:

- Studies that considered cognitive impairment and psychological sequelae (e.g., anxiety, depression, self-esteem, and quality of life impairments);
- Studies that considered alopecia;
- Studies focused on cancer-related side effects (e.g., cancer-related fatigue in patients not currently undergoing chemotherapy).

Regarding the management of chemotherapy side effects, the following papers were excluded:

- Studies on the benefits of nutrition or the use of extracts as a kind of treatment (e.g., mistletoe, Chinese herbal decoction);
- Studies on the acupuncture benefits.

Methodological Quality Assessment. Given the inherent challenges in conducting oncology studies, which frequently make certain quality assurance measures like blinding impractical, studies were not required to meet a predefined quality threshold for inclusion. However, their methodological quality was assessed by two independent researchers, using the National Institutes of Health (NIH) Checklist for Study Quality Assessment. The studies were evaluated based on affirmative responses (AR) to the checklists corresponding to the different types of studies. They were categorized as Poor (AR < 50%), Fair (50 ≤ AR > 75%), or Good (AR ≥ 75%) (Appendix A). The study was prospectively registered at the International Prospective Register of Systematic Reviews (PROSPERO) registry (CRD42023389064) (accessed on 15 January 2023). 

Categorization of the studies. Based on the selected studies, particular emphasis was placed on the most prevalent side effects. This included fatigue, reported by 74–85% of patients [7,10], nausea and vomiting in the first cycle affecting 79.3% of patients [10], and pain, diarrhea, and constipation, each reported by at least half of the patients [7,10]. Data were systematically extracted and organized using a table. The data for this systematic review was collected by two independent researchers, who each extracted data from the reports independently to minimize bias. Any discrepancies between the reviewers were resolved through discussion, and consensus was reached on all data points.

## 3. Results

**Figure 1:**
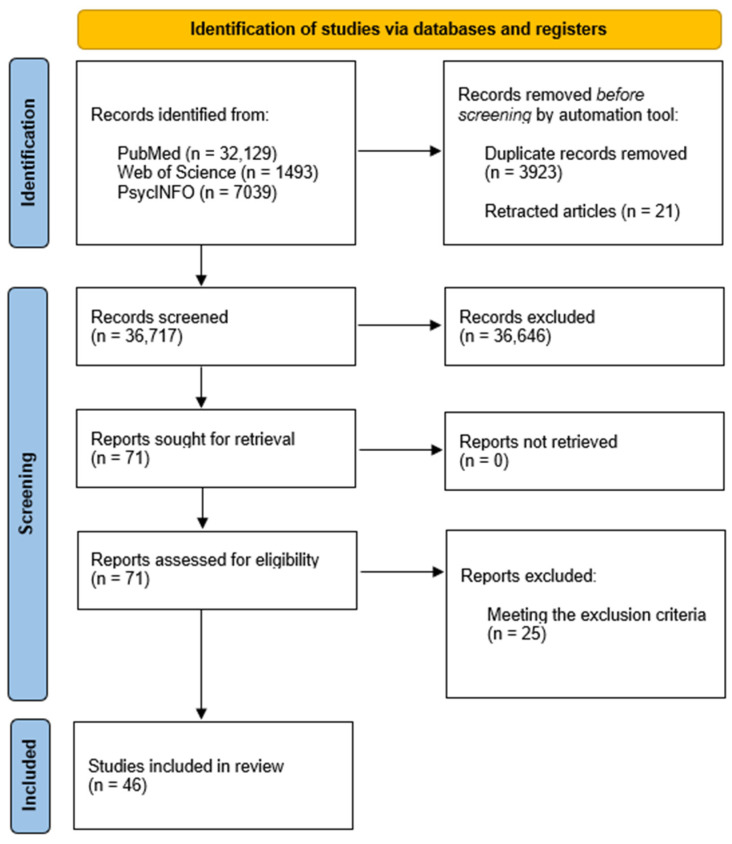
PRISMA flowchart summarizing study selection process.

**Studies Selection.** A total of 40,661 articles were identified through the search, with 21 retracted by authors and 3923 duplicates removed using Zotero software version 6.0.19 and version 7.0.3. In the first stage, two independent researchers (GP and FG) screened the titles and abstracts of the papers found. Specifically, the two researchers screened 36,717 articles. Simply by reading the titles, 36,305 papers were removed. After screening the abstract, another 341 articles were removed. Full-text screening of the remaining 71 studies resulted in the inclusion of 46 studies for the review (Figure 1). When discrepancies were identified between the two researchers, they discussed the article to reach a consensus. 

**Characteristics of the Studies.** The included studies were conducted globally, but not all specify the type of cancer affecting the patients examined. Among those that specify it, there are studies on patients with breast cancer (n = 14), prostate cancer (n = 1), lung cancer (n = 4), colorectal cancer (n = 3), stomach cancer (n = 1), gynecological cancer (n = 2), and acute myeloid leukemia (n = 2). Most studies included in this review are randomized control trials (n = 33), a few are pre-post studies (n = 6), and one is an observational cohort and cross-sectional study. 

We then categorized nonpharmacological interventions into major groups, namely mind-body interventions, educational interventions, physical interventions, psychological interventions, complementary and alternative therapies, and other interventions if the intervention did not fall into any of the previous categories.

Mind-body medicine includes techniques like guided imagery, progressive muscle relaxation, and yoga that enhance the mind’s impact on bodily function [23,24,25,26,27]. 

Educational interventions aim to help individuals manage side effects through guidance and knowledge, enhancing personal development and achieving educational goals [28,29]. Physical interventions involve tailored exercise programs to improve the quality of life and physical functions of chemotherapy patients [13]. Psychosocial care in oncology includes emotional support therapies like Cognitive Behavioral Therapy (CBT) and mindfulness-based approaches [30]. Complementary and alternative medicine encompasses practices outside conventional Western medicine, used by cancer patients to manage side effects, reduce stress, and engage in their care [31,32]. Regarding intervention categories, physical interventions, which include tailored exercise programs to improve the quality of life and physical functions of chemotherapy patients [13], were tested in 27.5% (n = 11) of studies. Mind-body interventions, encompassing techniques like guided imagery, progressive muscle relaxation, and yoga to enhance the mind’s impact on bodily function [23,24,26,27], were investigated in 26% (n = 12). Complementary and alternative medicine, including a variety of medical and healthcare practices outside the conventional Western medical framework [31], was studied in 17.5% (n = 7). Psychological interventions, such as emotional support therapies like Cognitive Behavioral Therapy (CBT) and mindfulness-based approaches [30], were examined in 12.5% (n = 5). Educational interventions, aimed at helping individuals manage side effects through guidance and knowledge, enhancing personal development, and achieving educational goals [28,29], were covered in 8.7% (n = 4). Other types of interventions were explored in 15% (n = 7) of the studies. Additional details are available in Table 1.

### 3.1. Fatigue

Twenty-eight studies have investigated fatigue as an outcome of nonpharmacological interventions [33,34,35,36,37,38,39,40,41,42,43,44,45,46,47,48,49,50,51,52,53,54,55,56,57,58,59,60].

#### 3.1.1. Mind-Body Interventions

Five studies investigated these interventions [42,48,49,52,59]; particularly, guided imagery proves effective in reducing fatigue both as a standalone intervention (*p* = 0.001) [52] and when combined with progressive muscle relaxation (*p* ≤ 0.0225) [42]. Progressive muscle relaxation seems to be effective also when combined with an early health intervention program (*p* < 0.01). Conversely, sensorimotor exercises, which integrate sensory input, such as coordination and balance with physical movement, seem ineffective in alleviating fatigue [49], as well as a program of Dru yoga (*p* > 0.05) [48]. On the other hand, a program of laughter yoga seems effective in reducing fatigue when control and intervention groups are compared (*p* = 0.001) [59].

#### 3.1.2. Educational Interventions

Only two studies have examined the efficacy of educational interventions to reduce fatigue [39,43]. The EASE (Energy and Sleep Enhancement) intervention, comprising three phone consultations with an oncology nurse aimed at enhancing patient understanding, appears ineffective in reducing fatigue (*p* > 0.05) [39]. However, symptom management education involving three educational sessions with a patient’s family member has shown promise in reducing both the intensity (*p* < 0.05) and interference (*p* < 0.05) of fatigue [43].

#### 3.1.3. Physical Interventions

Nine studies have investigated these interventions for chemotherapy-related fatigue [33,34,40,41,44,45,50,54,55]. Walking exercise programs have shown effectiveness, particularly in breast cancer patients who fully adhered to the program and for those with higher initial functional capacity (*p* < 0.01) [33]. In patients with acute myeloid leukemia, walking exercise seems to significantly improve fatigue metrics over time. Worst fatigue intensity improved from the first to the third week (*p* = 0.02; *p* = 0.05), average fatigue intensity improved from the first to the second week (*p* = 0.02; *p* = 0.01), and fatigue interference improved from the first to the third week (*p* = 0.04; *p* = 0.02) [36]. Additionally, the mean intensity of cancer-related fatigue in the 24 hours preceding the intervention was compared with the intensity measured on the fifth and tenth days after the intervention, showing a significant reduction on both days (*p* < 0.001) [50]. Walking interventions seem to be effective even when home-based, both at 6 weeks (*p* < 0.001) and 12 weeks (*p* < 0.01), with the intervention lasting 12 weeks starting from the first chemotherapy infusion [40]. Similarly, a structured and supervised 6-week intervention consisting of body-awareness training, massages, relaxation techniques, and resistance and fitness training yielded encouraging results (*p* = 0.003) [34].

However, a 12-week moderate-intensity exercise program, involving a combination of aerobic and resistance exercises, seems ineffective in alleviating fatigue among colorectal cancer patients (*p* = 0.079) [41]. Regarding the ideal duration and intensity of exercises, two studies emphasize the importance of engaging in at least 150 min of moderate to intense physical activity weekly (*p* = 0.01) [54]. This particularly benefits less physically active patients in reducing their physical exhaustion score [55]. Furthermore, exergaming sessions, which involve playing video games while engaging in physical movements, seem to effectively alleviate fatigue, with sustained efficacy observed throughout the entire 20-session protocol (*p* < 0.0001) [45].

#### 3.1.4. Psychological Interventions

Two studies investigating psychological therapies, specifically behavioral interventions, were included [35,38]. Neither study found statistically significant improvements in fatigue levels (*p* = 0.095; *p* > 0.05). This suggests that behavioral interventions may not be effective in reducing fatigue. However, one study did show a significant interaction between group and time, indicating greater improvement in physical fatigue for the treatment group (*p* = 0.03) [35], highlighting the need for further research.

#### 3.1.5. Complementary and Alternative Therapies

Four studies have explored the effectiveness of this kind of intervention [44,46,51,56]. Particularly, Qigong therapy seems to be effective in reducing chemotherapy-related fatigue, with a time-dependent effect (*p* < 0.001) [44] and a lower percentage of patients experiencing moderate-to-severe cancer-related fatigue (*p* < 0.01) [51]. Auricular acupressure also demonstrated significant results in reducing fatigue, both when used alone with Semen Vaccariae, the seed of the Vaccaria plant known as an herbal remedy (*p* < 0.01)) [56], and when combined with other interventions in therapeutic care (*p* < 0.01) [46].

#### 3.1.6. Other Interventions

Five studies [37,47,53,57,60] have demonstrated the effectiveness of various interventions. Specifically, nursing interventions, based on clinical knowledge and judgment, aimed at improving a patient’s health, preventing illness, and providing comfort and care seem to reduce both the prevalence (*p* < 0.01) and severity (*p* < 0.05) of fatigue when aimed at four self-management goals [47] and when based on a network positive psychological model (*p* < 0.001) [60]. However, interventions focused solely on symptom management or support were not effective in reducing fatigue prevalence and severity (*p* < 0.05) [53] across different time intervals (days 0–4, 5–8, 9–13) (*p* = 0.591; *p* = 0.579; *p* = 0.227) [37]. Additionally, watching nostalgic selected Disney movies during each chemotherapy session seems effective in reducing fatigue during chemotherapy in women with gynecological cancer (*p* = 0.01) [57].

### 3.2. Nausea and Vomiting

Twenty-five studies focused on nausea and vomiting as outcomes [34,37,40,42,43,44,47,48,52,58,61,62,63,64,65,66,67,68,69,70,71,72,73,74,75].

#### 3.2.1. Mind-Body Interventions

Ten studies consistently demonstrated the effectiveness of mind-body interventions in alleviating nausea and vomiting [42,48,52,58,61,63,66,67,69,70]. Guided imagery, when combined with music therapy, appears to effectively reduce pre-chemotherapy nausea after three cycles (*p* < 0.05) [67], as well as when coupled with progressive muscle relaxation after five cycles (*p* < 0.05) [63] in patients with breast or prostate cancer (*p* < 0.0001) [42]. Guided imagery alone also shows efficacy in reducing both nausea and vomiting severity (*p* = 0.001) [52], notably from the third chemotherapy cycle onwards, both for pre- and post-treatment symptoms (*p* = 0.0001) [69]. Progressive muscle relaxation seems to lead to a significant reduction in vomiting frequency within the initial four days following chemotherapy (*p* < 0.05) and in vomiting duration, although no notable difference in intensity was observed (*p* > 0.05). It also seems effective in alleviating vomiting frequency (*p* < 0.05) and duration (*p* = 0.016) [61]. This intervention also seems to be effective in reducing both nausea and vomiting when combined with an early health intervention program [58].

Furthermore, yoga appears effective in reducing post-chemotherapy nausea frequency (*p* = 0.01) and severity (*p* = 0.01) [66], particularly in cases of high symptom severity [70]. Moreover, yoga seems effective in reducing anticipatory nausea intensity (*p* = 0.003). Regarding vomiting, yoga shows efficacy in one study both for incidence (*p* = 0.01) and severity (*p* < 0.01) [70], and in one study only for anticipatory intensity (*p* = 0.04) [66]. Additionally, combining yoga with standard care can effectively manage nausea and vomiting over an extended period compared to standard care alone (*p* = 0.004) [48].

#### 3.2.2. Educational Interventions

One study demonstrated the efficacy of educational intervention, showing a significant reduction in both the frequency (*p* < 0.001) and severity (*p* < 0.05) of post-intervention nausea. Similarly, vomiting is also reduced in both frequency (*p* < 0.001) and severity (*p* < 0.05). However, no notable differences were found in anticipatory nausea and vomiting (*p* > 0.05) [43]. Another study involved three different groups where the first one received standard care, the second one received standard care with an educational intervention delivered through nursing specialists, and the third received the same educational intervention, but through a chatbot. Variance across all three arms of significant difference in symptom frequency and severity was found (*p* < 0.001). Moreover, when the first and third groups were compared, as well as the second and third groups, significant differences in distress levels were found (*p* < 0.001). However, no significant differences in distress were noted between the first and second groups (*p* = 1) [75].

#### 3.2.3. Physical Interventions

Four studies tested these interventions to reduce chemotherapy-related nausea and vomiting [34,40,41,64]. Effleurage massage, which involves circular stroking movements with the palm of the hand (*p* = 0.025) [64], and walking interventions at 6 weeks (*p* < 0.001) and 12 weeks (*p* < 0.01) seem to significantly decrease symptom severity and interference at 6 weeks (*p* = 0.02) [40]. Conversely, both a planned and supervised 6-week program involving body-awareness training, massages, relaxation techniques, and resistance and fitness training (*p* > 0.05) [34] and supervised exercise programs exhibit no significant effects on nausea or vomiting (*p* = 0.385) [41].

#### 3.2.4. Psychological Interventions

Three studies have examined the effectiveness of these interventions [62,65,71], concluding that cognitive-behavioral interventions appear to work in reducing symptom severity after 10 and 20 weeks (*p* < 0.01) [62]. Pure behavioral interventions also seem effective in determining the severity of nausea at the midpoint (*p* = 0.01) [71]. Instead, group psychotherapy appears effective primarily for vomiting episodes (*p* < 0.05) [65].

#### 3.2.5. Complementary and Alternative Therapies

Three articles in this review focus on complementary and alternative medicine [44,68,72]. Acupressure did not demonstrate significant results in reducing nausea and vomiting (*p* = 0.80) [68]. Conversely, the Qigong intervention significantly improved nausea and vomiting in 21 days (*p* < 0.001) [44]. Regarding foot reflexology, while it showed no significant difference in vomiting (*p* = 0.99) or frequency of delayed nausea (*p* = 0.28), it did lead to reduced antiemetic use (*p* = 0.04) [72].

#### 3.2.6. Other Interventions

Two studies have shown discouraging results for nursing interventions in reducing nausea, but seem promising in reducing vomiting [37,47]. Indeed, the CHEMO-SUPPORT intervention, a personalized nursing intervention targeting four self-management objectives, appears ineffective in reducing both the severity (*p* = 0.24) and prevalence (*p* = 0.41) of nausea [47]. Similarly, the WISECARE+ intervention, administered by nurses with recommended actions based on the severity of the patient’s symptoms, does not appear to alleviate nausea (*p* = 0.290), but it does yield promising results for reducing vomiting (*p* = 0.041) [37]. On the other hand, a nursing intervention based on risk assessment seems effective in reducing delayed nausea level (*p* = 0.006) and delayed vomiting frequency (*p* = 0.027), while seemingly ineffective for acute symptoms (nausea: *p* = 0.91; vomiting: *p* = 0.146). One study investigated the efficacy of virtual reality in alleviating nausea, but it also failed to identify significant changes in nausea levels between intervention and control groups, both before (*p* = 0.42) and following chemotherapy (*p* = 0.39) [73].

### 3.3. Pain

In this review, sixteen studies focused on pain as a primary outcome [29,34,39,40,42,43,44,45,47,52,53,58,59,62,73,76,77].

#### 3.3.1. Mind-Body Interventions

The efficacy of guided imagery in addressing chemotherapy-related pain has been investigated in two studies [42,52]. Combining guided imagery with progressive muscle relaxation training significantly reduces pain levels (*p* = 0.0003) [42], and guided imagery alone also appears to contribute to pain improvement (*p* = 0.001) [52]. Regarding progressive muscle relaxation, it seems to be ineffective in reducing pain when combined with an early health intervention program [58]. Finally, a program of laughter yoga seems effective in reducing pain when control and intervention groups are compared (*p* = 0.001) [59].

#### 3.3.2. Educational Interventions

Three studies have examined the efficacy of these interventions to reduce pain [29,39,43]. The EASE (Energy and Sleep Enhancement) intervention, comprising three telephone consultations with an oncology nurse aimed at improving the patient’s comprehension, appears effective in reducing pain and functional interference, specifically among unemployed patients (*p* < 0.05), who benefit more from the intervention compared to those who continued working during cancer treatments [39]. Conversely, symptom management consisting of three educational sessions seems to be ineffective in reducing either the frequency (*p* > 0.05) or severity (*p* > 0.05) of pain [43]. Additionally, pairing a pain consultation with a pain education program outperforms standard care, resulting in significantly lower average (*p* = 0.03) and current pain levels (*p* = 0.016) and reduced interference of pain with daily activities (*p* = 0.016) [29].

#### 3.3.3. Physical Interventions

Five studies have suggested that physical interventions seem to be effective in reducing chemotherapy-related pain [34,40,41,54,77]. A structured and supervised program lasting 6 weeks, which included body-awareness training, massages, relaxation techniques, and resistance and fitness training, showed a decreasing trend in myalgia (*p* = 0.013) and pain (*p* = 0.013) levels within 6 weeks [34], as well as in peripheral neuropathy immediately after the intervention (*p* = 0.028) and at 4 weeks (*p* = 0.031) [77]. Similarly, walking intervention appears effective in reducing pain severity at 6 weeks (*p* < 0.001) and 12 weeks (*p* < 0.01) [40]. Pain interference also decreased at 6 weeks (*p* = 0.02). Finally, a 12-week moderate-intensity exercise regimen, including both aerobic and resistance exercises [41], also demonstrated efficacy after 3 months (*p* = 0.02). This result is consistent with another study, where patients were engaged for at least 150 min of moderate to intense physical activity weekly, and it seems to alleviate pain (*p* = 0.01) [54].

#### 3.3.4. Psychological Interventions

One study considered cognitive-behavioral intervention, demonstrating its efficacy in reducing pain among patients with elevated baseline scores at both 10 and 20 weeks (*p* < 0.01) [62].

#### 3.3.5. Complementary and Alternative Therapies

Two studies investigated the efficacy of complementary and alternative therapies in pain reduction [44,76]. Reiki therapy, a complementary practice involving a practitioner using hand placements on or near the body to channel energy, aims to promote relaxation, reduce stress, and enhance the body’s natural healing processes. It appears effective for patients who underwent the full cycle (*p* < 0.0191), especially after the initial three sessions [76]. The Qigong intervention also seems to work within the first 21 days of intervention (*p* < 0.001) [44].

#### 3.3.6. Other Interventions

Two studies examined the effectiveness of nursing interventions, yielding conflicting results [47,53]. Specifically, an individually tailored nursing intervention appears effective in reducing pain severity (*p* < 0.05) [47]. In contrast, standard oncology care supplemented with proactive telephone nursing guidance and support does not produce similar promising outcomes, neither for the number of symptoms (*p* = 0.80) nor symptoms of distress (*p* = 0.86) [53]. One study investigated the efficacy of virtual reality in alleviating pain, but it did not find significant changes between intervention and control groups, both before (*p* = 0.69) and following chemotherapy (*p* = 0.26) [73].

### 3.4. Diarrhea and Constipation

Thirteen studies within this review aimed to alleviate chemotherapy-related diarrhea and constipation with nonpharmacological treatments [34,40,42,43,44,47,48,53,54,58,59,62,75].

#### 3.4.1. Mind-Body Interventions

Four studies within this category were identified [42,48,58,59]. While guided imagery combined with progressive muscle relaxation seems to alleviate diarrhea (*p* < 0.0001), it is associated with an increase in symptoms of constipation (*p* < 0.0001) [42]. Neither progressive muscle relaxation combined with an educational program seems effective in reducing diarrhea [58]. Additionally, yoga interventions did not demonstrate effectiveness in reducing gastrointestinal symptoms when implemented as a Dru yoga program (*p* > 0.05) [48] or when implemented as a laughter yoga program (diarrhea: *p* = 0.650; constipation: *p* = 0.321) [59].

#### 3.4.2. Educational Interventions

One study investigated the effects of three planned educational sessions on chemotherapy patients, observing no statistically significant impact on the frequency (*p* > 0.05) or severity (*p* > 0.05) of diarrhea and constipation [43]. In a different study, three groups were assigned to receive different treatments, including standard care for the first group, standard care with an educational intervention from nursing specialists for the second group, and the same educational intervention for the third group via a chatbot. There was significant variation in the frequency and intensity of symptoms across all three arms of a significant difference (*p* < 0.001). Furthermore, significant differences in distress levels were observed (*p* < 0.001) when comparing the first and third groups, as well as the second and third groups; no significant differences in distress were observed (*p* = 1) between the first and second groups [75].

#### 3.4.3. Physical Interventions

Three studies on physical therapies were included, revealing various findings [34,41,54]. Despite physically inactive patients showing higher diarrhea scores compared to active ones (*p* = 0.04) [54], neither a 6-week structured and supervised program comprising various techniques (*p* = 0.60) [34] nor a 12-week moderate-intensity exercise program appears to reduce constipation (*p* = 0.62) or diarrhea (*p* = 0.787) effectively [41].

#### 3.4.4. Psychological Interventions

One study examined behavioral intervention’s efficacy in reducing diarrhea and constipation, yielding encouraging results. Notably, this intervention significantly reduced symptom severity at both 10 and 20 weeks (*p* < 0.01) [62].

#### 3.4.5. Complementary and Alternative Therapies

Only one study examined the effectiveness of complementary and alternative therapies. Specifically, a Qigong intervention appears to effectively reduce diarrhea and constipation within 21 days (*p* < 0.001) [44].

#### 3.4.6. Other Interventions

Two studies considered nursing interventions, but none found encouraging results [47,53]. The CHEMO-SUPPORT treatment, a personalized nursing intervention targeting four self-management objectives, appears ineffective in addressing both diarrhea (*p* = 0.77) and constipation (*p* = 0.11) [47]. Standard oncology care supplemented with proactive telephone nursing guidance and support also fails to yield promising outcomes, both for the number of symptoms (*p* = 0.80) and distress (*p* = 0.86) [53].

## 4. Discussion

Chemotherapy, while crucial in cancer treatment, frequently induces severe physical side effects, significantly impacting patients’ quality of life. To mitigate these effects, nonpharmacological interventions have been investigated as adjuncts to standard care, aiming to enhance patient well-being during treatment. This review systematizes research on nonpharmacological treatments for prevalent chemotherapy physical side effects.

Although mind-body interventions are among the most effective types of treatments, not all of them are successful. Guided imagery has shown efficacy in mitigating chemotherapy-induced physical side effects, such as fatigue, nausea, vomiting, and pain, in two studies [52,69]. Notably, when integrated with music therapy, guided imagery maintains its effectiveness in managing pre-chemotherapy nausea and vomiting [67]. Additionally, when combined with progressive muscle relaxation, it proves effective in managing fatigue, nausea, vomiting, pain, and diarrhea. However, it may have less impact on alleviating constipation [42]. Progressive muscle relaxation indeed seems to help with various side effects, but no firm conclusions can be drawn on its effectiveness since it is often combined with other interventions [42,58,63].

The mechanisms underlying the effectiveness of these interventions are multifactorial. Guided imagery emerges as a promising intervention for alleviating chemotherapy challenges by reducing stress and anxiety, thereby improving overall well-being [69,78]. Psychological factors such as optimism play a protective role in maintaining physical health during cancer treatment [79]. Guided imagery probably helps manage side effects by redirecting attention from discomfort to pleasant mental imagery, serving as a constructive distraction during treatment [52]. It also helps cope with treatment demands, promoting feelings of control and empowerment [42,80]. Regarding mind-body interventions, combining sensory and motor inputs with physical movements has not been proven effective in alleviating side effects, particularly fatigue [49]. This could be due to significant individual variability and the small sample size (n = 36), which reduced statistical power and hindered the detection of significant results.

Yoga does not appear to be effective in managing diarrhea and constipation [48,59], but returned mixed results regarding fatigue [48,59]. It is important to consider that the measurement of fatigue was subject to significant individual variances, and the interventions in the studies were different. On the other hand, yoga emerges as a promising intervention for alleviating nausea [66,70] and vomiting [48,70], probably because it can influence the autonomic nervous system, helping to restore the balance between the sympathetic nervous system and the parasympathetic nervous system [81].

The effectiveness of educational interventions for managing fatigue in oncology patients presents uncertainty due to conflicting study results [39,43]. It is imperative to carefully evaluate the components and delivery methods of the interventions to optimize fatigue management strategies. Patient education and family involvement may play a significant role in comprehensive symptom management programs. However, the efficacy of educational interventions in pain management remains uncertain as research findings are mixed [29,39,43]. These variations could stem from the specific cancer types targeted by the interventions, necessitating cautious interpretation. Concerning diarrhea and constipation management, three planned interventions were found ineffective, albeit limited to patients with hematological cancers [43], while an educational program delivered by a ChatBot seems to help [75]. Educational interventions appear promising in mitigating post-chemotherapy nausea and vomiting [43,75], potentially attributed to patients’ enhanced understanding and experience in symptom management [82].

Physical interventions do not appear effective in reducing chemotherapy-related nausea, vomiting [34,41], diarrhea, and constipation [34,41], despite one study showing that diarrhea may be exacerbated by physical inactivity [54]. This lack of effectiveness may be influenced by various factors, including advancements in antiemetic treatments for nausea and limitations in study designs, such as biases in patient selection and missing data points.

Regarding walking interventions, they have shown potential benefits for controlling fatigue [33,36,40,50], pain [40], nausea, and vomiting [40], emphasizing the importance of tailoring exercises based on the clinical story of the patient. The correlation between increased physical activity levels and reduced symptom distress and severity is evident, suggesting that being more physically active may lead to fewer and less severe symptoms. Additionally, physical activity has been linked to improvements in mood disturbance, potentially associated with enhanced physical functioning [36,40,50].

Another intervention that seems to reduce nausea and vomiting is effleurage massage [64]. However, operator training and standardization are essential, and further research should focus on these variables to draw more robust conclusions.

Other physical interventions structured on diverse protocols also seem effective in alleviating fatigue [34,54,55] and pain [34,41,77]. The effectiveness of the exergaming protocol on fatigue [45] is promising.

Physical intervention’s effectiveness is attributed to its ability to enhance overall physical health through increased oxygen flow, endorphin release, and improved energy levels [35,83]. Furthermore, physical activity triggers the release of neurotransmitters, improving mood and overall well-being [33,36]. Depression and physical symptoms are often correlated; as depressive symptoms decrease, physical symptoms tend to decrease as well [84]. Additionally, group walking interventions facilitate social interaction, combating isolation, and providing emotional support [50,77]. Studies suggest that greater social and emotional support correlates with a higher quality of life, mainly when positive interactions occur with significant individuals, including healthcare professionals [85,86].

Psychological interventions, such as behavioral interventions, seem helpful for nausea, vomiting [62,71], pain [62], diarrhea, and constipation [62], probably because they refer to problem-solving abilities that engage patients in specific intervention strategies designed to reduce their symptom burden. Conversely, they exhibited limited effectiveness for fatigue [35,38] necessitating larger-scale studies with adequate statistical power and more heterogeneous samples in terms of baseline fatigue level. Group psychotherapy has been investigated only in one study [65] for nausea and vomiting. Their effectiveness likely stems from enhancing patients’ knowledge about their symptoms within a supportive social context. This approach values emotions as a source of closeness, fostering a better understanding and management of symptoms through improved problem-solving abilities and emotional support [87].

Qigong, an alternative and complementary medicine intervention, seems promising for every side effect considered in this review [44,51]. This is likely because calming the mind and body helps cancer patients better manage the physical and emotional challenges of treatment [51]. It also strengthens the immune system, counteracting the weakening effects of chemotherapy and promoting homeostasis of the autonomic nervous system. Additionally, Qigong’s meditative aspects can improve mental well-being by reducing symptoms of anxiety and enhancing overall mood [51]. The same explanation could be extended to the effectiveness of Reiki therapy for pain [76].

Acupressure interventions also seem effective in alleviating fatigue [41,46] but do not yield the same result for nausea and vomiting [68]. These symptoms do not seem to benefit from reflexology interventions either [72]. However, it must be noted that studies focused on complementary and alternative medicine are difficult to generalize because outcomes may vary depending on the practitioner and family members involved. Indeed, the comprehensive analysis suggests that the proficiency and emotional support provided by caregivers or family members significantly influence treatment outcomes. Indeed, higher perceived support predicts better physical functioning, particularly in the context of chemotherapy [85]. Moreover, the effectiveness may depend heavily on the patient’s beliefs and preferences [88].

Nursing interventions provide various results for fatigue [37,47,53,60], nausea, vomiting [37,47,74], and pain [47,53]. To comprehend these findings, it is crucial to recognize that healthcare providers’ clinical expertise and interpersonal abilities might impact treatment response. Thus, the nurse’s attributes could be a confounding factor not thoroughly explored in the referenced studies. On the other hand, nursing interventions show no efficacy for diarrhea and constipation [47,53], but again generalization is complex.

Watching Disney films during chemotherapy [57] demonstrated encouraging results, albeit limited to gynecological cancer patients. Disney movies could provide emotional and social benefits since they can distract patients providing a sense of hope and nostalgia. Individual patients’ personal preferences and childhood experiences may influence the effectiveness of such interventions, warranting further investigation.

Finally, a virtual reality intervention seems ineffective in reducing pain and nausea, and this can be attributed to the sample of patients under investigation, all of whom suffer from lung cancer, which is characterized by more severe and complex symptoms than other types of cancer [73].

To resume, interventions consistently effective across multiple studies include guided imagery, educational interventions, physical activities, alternative therapies like Qigong, and nurse-administered strategies. The studies reviewed offer varied insights into intervention efficacy. However, all of them seem to rely on a mechanism to reduce anxiety and stress, inducing a state of greater emotional well-being. The gut plays an important role in the mind-body relationship, which has a vital role in the body’s immune system. Emotional states, such as stress, anxiety, and depression, have direct effects on the gastrointestinal system. Stress, for instance, can trigger the release of stress hormones like cortisol, which can disrupt gut function and lead to symptoms like abdominal discomfort, diarrhea, or constipation [89]. Indeed, psychological stress can weaken the immune response in the gut, increasing vulnerability to infections and inflammation [90]. Concerning chemotherapy side effects, distress and anxiety experienced during chemotherapy could exacerbate them, especially nausea, vomiting, diarrhea, and constipation. Additionally, it is plausible to suggest that the efficacy of certain nonpharmacological interventions on gastrointestinal issues depends on their ability to stimulate the parasympathetic nervous system, which regulates digestion and intestinal function [91].

The nociceptive system also appears to play a fundamental role in the physiological relationship between mind and body, particularly in pain perception. Emotional and cognitive factors can significantly modulate the perception of pain. For example, stress, anxiety, fear, and depression can amplify the perception of pain, making it feel more intense and distressing. Conversely, positive emotions, distraction, relaxation, and certain cognitive techniques can help reduce pain perception [92].

## 5. Conclusions

In conclusion, this review highlights the diverse range of nonpharmacological interventions for managing chemotherapy-induced side effects. While some interventions show promise in addressing common symptoms, others have inconclusive findings, necessitating further investigation to establish their efficacy definitively. A significant challenge in drawing definitive conclusions stems from the considerable variability among the studies reviewed, both in the types of interventions and the specific protocols employed. To achieve more reliable and replicable results, future research should adopt standardized protocols and replicate studies with larger sample sizes.

Additionally, the studies included exhibited significant variation in sampling, both in terms of sample size and patient characteristics. Often, studies focus on patients with a single type of cancer, limiting the generalizability of the findings. Greater homogeneity in samples and a broader representation of different cancer types and stages are necessary to enhance the validity and applicability of the results.

Nonetheless, the importance of customizing interventions and integrating pharmacological approaches with nonpharmacological ones to provide holistic therapy to the patient remains clear. Guided imagery stands out for its accessibility, simplicity, and customization potential, making it suitable for integration into chemotherapy cycles [67,93]. Walking interventions offer a safe form of exercise adaptable to individual fitness levels and needs, with low impact on joints and muscles [94]. Psychological interventions improve emotional well-being and are tailored to each patient’s unique requirements [95]. Alternative therapies, like Qigong, are gaining interest due to their non-invasiveness and customization possibilities [96]. Nurse-administered interventions benefit from nurses’ strong patient relationships and adaptability to individual needs [47]. While these interventions show promise in oncology for their accessibility, safety, and tailoring, their feasibility in clinical practice depends on patient preferences and healthcare resources.

The present review systematically synthesizes heterogeneous research on nonpharmacological interventions over the past 20 years, promoting a holistic approach to cancer patient care. It emphasizes the importance of prioritizing patient well-being and tailoring treatment plans accordingly. However, limitations such as the potential inadvertent exclusion of relevant studies and the generally low methodological quality in research on nonpharmacological interventions are noted. The need for future research standardization and rigorous methodologies is emphasized, given the challenges posed by heterogeneous study designs and outcomes that precluded the conduct of a meta-analysis.

Recommendations for future research include ensuring methodological quality, considering patient preferences and beliefs, and examining practitioner skills’ impact. Objective variables should be included in symptom evaluation and standardized, validated measures should be used to minimize subjectivity and bias. Psychological variables’ role in symptom reporting must be comprehensively measured to obtain an accurate representation. Thus, future research should address these factors to improve intervention effectiveness assessment and comparability across studies.

In summary, although preliminary indications suggest the effectiveness of certain nonpharmacological interventions in reducing chemotherapy side effects, further research with rigorous methodologies is needed to confirm these findings and expand their clinical applicability.

## Figures and Tables

**Table 1:** Results of the studies.

Article	Intervention	Structure	Sample	Measurement	Effectiveness
[29]	Pain consultation (PC) and Pain education program (PEP)	PC entails a pain assessment by a neurooncologist in addition to a thorough physical and anamnestic examination of the patient. PEP intervention included the use of multiple teaching methods, which were provided both in the outpatient clinic and by telephone.	72 cancer patients with nociceptive cancer-related pain	Brief Pain Inventory (BFI)	**Pain** Average intensity: control group vs. experimental group (mΔPI = 1.13 vs. 1.95) (20% vs. 31%; *p* = 0.03). Current intensity: control group vs. experimental group (mΔPI = 0.67 vs. 1.50) (16% vs. 30%; *p* = 0.016). Daily interference: mean reduction control group vs. experimental group (0.11 vs. 0.91) (2.5% vs. 20%; *p* = 0.01).
[33]	Physical exercise.	Walking five to six times per week in the desired heart rate range (about 50 to 70% of maximum heart rate) at a moderate pace for six weeks. Walking for 15 min quickly escalated to 30 min as the training went on.	119 women with breast cancer (stage 0–III).	Piper Fatigue Scale (PFS).	**Fatigue**: Change between baseline and post-test for low walkers = 2.05 (SD = 2.84) (*p* < 0.01).
[34]	Multidimensional exercise program.	A structured, monitored intervention lasting 6 weeks, encompassing body-awareness training, massage therapy, relaxation techniques, and resistance and fitness training.	54 cancer patients.	Semi-structured diaries.	**Fatigue** Treatment-related: from 0.83 to 0.55 (*p* = 0.003). **Gastrointestinal symptoms**: from 0.57 to 0.30 (*p* = 0.60). **Myalgia**: from 0.36 to 0.17 (*p* = 0.013) **Pain**: from 0.53 to 0.39 (*p* = 0.041). **Nausea**: from 0.22 to 0.24. **Vomiting**: from 0.04 to 0.04.
[35]	Behaviorally oriented intervention.	Three individual, face-to-face, 60-min sessions at 3 to 4 weekly intervals (coinciding with the administration of chemotherapy).	55 cancer patients.	Visual Analogue Scale for Fatigue (VAS-F); Fatigue Outcome Measure (FOM)—specifically designed for this study; Multidimensional Fatigue Inventory (MFI).	**Fatigue** VAS: coefficient = 8.9, 95% CI [21.6–19.4] (*p* = 0.095). Physical (MFI) = interaction between group and time: T2 and T3 (coefficient, 1.6; 95% CI, 0.1–3.0; *p* = 0.03).
[36]	Walking intervention.	12 min of walking in the hospital hallway, five days a week for three weeks.	24 hospitalized acute myelogenous leukemia patients.	Brief Fatigue Inventory (BFI).	**Fatigue** Worst fatigue intensity: Significant differences from the end of the first week (z = −2.31) (*p* = 0.02) to the end of the third week (z = −1.95) (*p* = 0.05). Average fatigue intensity: Significant differences from the end of the first week (z = −2.36) (*p* = 0.02) to the end of the second week (z = −2.50) (*p* = 0.01). Fatigue interference: Significant differences from the end of the first week (z = −2.03) (*p* = 0.04) to the end of the third week (z = −2.28) (*p* = 0.02).
[37]	WISECARE+	Evidence-based nursing practice protocol that recommended actions based on the severity of the symptoms. The symptom assessment was used in conjunction with a structured and cyclical symptom assessment that was completed by the patients themselves. Mean symptom scores were calculated by taking the average of the individual daily score (14 days) per patient.	249 cancer patients.	Chemotherapy Symptom Assessment Scale (C-SAS).	**Fatigue** No evidence of a difference in fatigue levels between the pre- and post- intervention groups at any of days 0–4, 5–8, or 9–13 (z = 0.5, *p* = 0.591; z = 0.5, *p* = 0.579; z = 1.2, *p* = 0.227, respectively). **Vomiting** Considering both time and group effects simultaneously, there was a significant decrease in vomiting levels post-intervention at all time points (F(1,235) = 4.2; *p* = 0.041). **Nausea** Considering both time and group effects simultaneously, there was no evidence of an effect on nausea levels at any time point (F(1,235) = 1.1; *p* = 0.290).
[38]	Behavioral therapy intervention.	The intervention included modified stimulus control, modified sleep restriction, relaxation therapy, and sleep hygiene.	219 cancer patients.	The Piper Fatigue Scale (PFS).	**Fatigue** (*p* > 0.05). Before treatment: 4.05 (±2.1). 30 days after treatments: 4.07 (±2.4).
[39]	Energy and Sleep Enhancement (EASE) intervention.	Three phone consultations with an oncology nurse during the second, third, and fourth weeks following the initial therapy. An interactive delivery method was used to deliver the intervention, building on the individual’s prior understanding of energy-saving techniques, sleep hygiene, and his or her symptom response.	276 cancer patients.	General Fatigue Scale (GFS); Brief Pain Inventory (BPI).	**Fatigue**. GFS Experimental: from 5.19 (2.14) to 4.89 (1.92). Control: from 5.12 (2.05) to 4.82 (2.03). Study group by time: F-test = 0.06 (*p* > 0.05). **Pain.** BPI Experimental: from 1.99 (2.16) to 2.27 (2.26). Control: from 1.7 (2.14) to 2.15 (2.25) Study group by time: F-test = 0.32 (*p* > 0.05).
[40]	Home-based walking program.	Participants exercised for the duration of their chemotherapy treatment, beginning 2 or 3 days after starting each chemotherapy cycle. The exercise participants were asked to walk briskly three times per week for 12 weeks concurrently with adjuvant chemotherapy. Each exercise session included 5 min of warm-up, 30 min of moderate-intensity brisk walking (60–80% of age-adjusted maximal heart rate), and 5 min of cool down.	40 women with breast cancer (postoperative stage I–III A).	The Taiwanese Version of the M.D. Anderson Symptom Inventory (MDASI-T).	The exercise participants reported significantly lower symptom severity than the control group at 6 weeks (F = 10.59, *p* < 0.001) and 12 weeks (F = 9.04, *p* < 0.01). Additionally, exercise participants reported significantly less symptom interference than the control group at 6 weeks (F = 6.67, *p* = 0.02).
[41]	Supervised exercise program.	Participants in the supervised-exercise group followed a 12-week program consisting of moderate-intensity aerobic and resistance exercises. Each week, they attended two 40–60 min sessions guided by a research assistant. The program included a 5-min warm-up, 30–40 min of combined aerobic and resistance exercises using dumbbells and sandbags, and a 5-min cool-down. The exercise intensity gradually increased from 40% to 55% of the maximum heart rate, with session length and intensity adjusted based on individual progress and ability.	45 patients with colorectal cancer (stage II or III).	14-item Fatigue Symptom Inventory (FSI); Standard Chinese version of the European Organization for Research and Treatment of Cancer Quality of Life Core Questionnaire (EORTC-QLQ-C-30).	**Fatigue:** *p* = 0.079, partial ƞ^2^ = 0.07. **Pain:** *p* = 0.02, partial ƞ^2^ = 0.12. **Nausea and vomiting:** *p* = 0.385, partial ƞ^2^ = 0.018. **Diarrhea:** *p* = 0.787, partial ƞ^2^ = 0.002. **Constipation:** *p* = 0.620, partial ƞ^2^ = 0.006.
[42]	Guided Imagery and Progressive Muscle Relaxation.	Four weekly supervised sessions of GI and PMR and daily unsupervised sessions. The intervention consisted of a 2-min breathing exercise, a 10-min activity for progressive muscular relaxation, and a 15-min session for pleasant, guided imagery.	208 patients with clinical diagnosis of breast (clinical stage, T3N1M0) or prostate cancer (clinical stage T3a, Gleason score ≥ 8).	Cancer Fatigue Scale (CFS); The European Organization for Research and Treatment of Cancer Quality of Life Core Questionnaire (EORTC-QLQ-C-30); Numeric pain scale. Revised Rhodes index of nausea, vomiting, and retching (INVR)	**Fatigue:** from 27.6 (SD = 3.9) to 19.3 (SD = 3.5) (*p* < 0.0225). **Diarrhea:** −1.5 (Mean change—*p* Paired *t*-test) (*p* < 0.0001). **Constipation:** +0.8 (Mean change—*p* Paired *t*-test) (*p* < 0.0001). **Pain:** from 4.17 (SD = 2.5) to 2.5 (SD = 1.6) (*p* = 0.0003). **Nausea and vomiting:** from 25.4 (SD = 5.9) to 20.6 (SD = 5.6) (*p* < 0.0001).
[43]	Symptom management intervention.	Three educational sessions were provided: the first before the initial chemotherapy cycle, covering symptoms, causes, prevention, and control; the second 35–45 days later, focusing on symptom management; and the third before the third chemotherapy cycle. Each session included a family member of the patient. Patients were also informed they could contact the researcher directly. Due to ethical considerations, the control group received their educational session after those given to the experimental group, with data collected from both groups.	140 cancer patients.	Chemotherapy Symptom Assessment Scale (CSAS).	**Fatigue** Frequency: control group pre-post intervention (92.1%–92.1%) vs. experimental group pre-post intervention (88.4%–28.2%) (*p* < 0.05). Severity: control group pre-post intervention (2.68 ± 0.43; 2.88 ± 0.25) vs. experimental group pre-post intervention (2.34 ± 0.45; 1.08 ± 0.35) (*p* < 0.05). **Diarrhea** Frequency: control group pre-post intervention (85.4%–93.3%) vs. experimental group pre-post intervention (88.7%–96.6%) (*p* > 0.05). Severity: control group pre-post intervention (1.71 ± 0.45; 1.89 ± 0.58) vs. experimental group pre-post intervention (1.76 ± 0.41; 1.78 ± 0.43) (*p* > 0.05). **Constipation** Frequency: control group pre-post intervention (84.7%–95.2%) vs. experimental group pre-post intervention (96.7%–95.5%) (*p* > 0.05). Severity: control group pre-post intervention (1.31 ± 0.55; 1.36 ± 0.68) vs. experimental group pre-post intervention (1.56 ± 0.52; 1.57 ± 0.52) (*p* > 0.05). **Pain** Frequency: control group pre-post intervention (74.5%–91.7%) vs. experimental group pre-post intervention (93.3%–95.7%) (*p* > 0.05). Severity: control group pre-post intervention (1.32 ± 0.47; 1.42 ± 0.25) vs. experimental group pre-post intervention (1.52 ± 0.52; 1.62 ± 0.40) (*p* > 0.05).
[44]	Chan Chuang Qigong.	Initial instructions on Chan-Chuang qigong during the participants’ first 2-day hospital admission for chemotherapy, and successive 21-day at-home exercises guided by a nursing booklet on Chan-Chuang qigong.	100 cancer patients with non-Hodgkin lymphoma.	Fatigue: Brief fatigue inventory-Taiwan form (BFI-TF); Current fatigue during the past 24 h; Visual Analogue Scale (VAS); a Chinese version of the EORTC QLQ-C30 (European Oorganization for Rresearch and tTreatment of Ccancer quality of life questionnaire).	**Fatigue** Intensity: baseline—day 21 change = 5.12 (SD = 1.62) (t = −13.69; *p* < 0.001). Interference: baseline—day 21 change = 5.33 (SD = 1.49) (t = −16.25; *p* < 0.001). **Diarrhea**: baseline—day 21 change = 6.28 (SD = 14.84) (*p* < 0.001). **Constipation:** baseline-day 21 change = 43.75 (SD = 18.39) (*p* < 0.001). **Pain**: baseline—day 21 change = 22.57 (SD = 14.78) (t = −10.46; *p* < 0.001). **Nausea and vomiting**: baseline—day 21 change = 14.24 (SD = 22.01) (t = −4.67; *p* < 0.001).
[45]	Exergaming.	Exergaming is the combination of exercise and play and consists of playing video games while performing physical movements. The activity lasted for 20 sessions.	45 cancer patients (excluded: patients with stage IV cancer).	Functional Assessment of Chronic Illness Therapy Fatigue (FACIT-F) questionnaire.	**Fatigue**: ANOVA-P (0.0025) (*p* < 0.0001).
[46]	Therapeutic care.	Acupressure at bilateral Hegu (LI4, in the middle of the second metacarpal bone on the radial side), Zusanli (ST36, 3 cun below the lower border of the patella, 1 finger width lateral from the anterior border of the tibia), and Sanyinjiao (SP6, 3 cun directly above the tip of the medial malleolus on the posterior border of the tibia), 30 min/d, each point 10minutes, 3 days weekly for 12 weeks.	48 women with breast cancer.	Multidimensional fatigue inventory (MFI).	**Fatigue**: difference between groups. General Fatigue Week 6: −2.0 (−2.8, −1.7) (*p* < 0.01). Week 12: −3.1 (−3.9, −2.3) (*p* < 0.01). Physical Fatigue Week 6: −1.8 (−2.6, −1.5) (*p* < 0.01). Week 12: −3.2 (−4.0, −2.6) (*p* < 0.01).
[47]	Nursing intervention.	CHEMO-SUPPORT is a tailored nursing intervention designed to achieve four self-management objectives: practicing preventive self-care, monitoring and reporting symptoms, timely communication with healthcare professionals, and performing self-care to alleviate symptoms. The program includes an initial in-person coaching session, a follow-up telephone coaching session, a patient education pamphlet, and access to an online or on-call nurse service, with additional coaching provided if necessary.	143 cancer patients.	Overall symptom.	**Fatigue** Prevalence: OR = 2.8; 95% CI [1.4, 5.9] p(per group effect) = 0.00. Severity: OR = 2.3; 95% CI [1.4, 3.9] p(per group effect) = 0.00. **Diarrhea** Prevalence: OR = 0.9; 95% CI [0.5, 1.9] p(per group effect) = 0.84. Severity: OR = 1; 95% CI [0.5, 1.9] p(per group effect) = 0.98. **Constipation** Prevalence: OR = 1.2; 95% CI [0.7, 2.3] p(per group effect) = 0.5. Severity: OR = 1.1; 95% CI [0.6, 2.1] p(per group effect) = 0.7. **Pain** Prevalence: OR = 1.4; 95% CI [0.8, 2.6] p(per group effect) = 0.26. Severity: OR = 2.1; 95% CI [1.2, 3.6] p(per group effect) = 0.01. **Nausea** Prevalence: OR = 1.3; 95% CI [0.7, 2.2] p(per group effect) = 0.41. Severity: OR = 1.4; 95% CI [0.8, 2.3] p(per group effect) = 0.24.
[48]	Yoga.	The yoga program involved weekly 75-min sessions at the hospital over 12 weeks, starting 1–2 weeks before chemotherapy. Based on Dru Yoga, the program was designed to reduce fatigue and improve quality of life for women with breast cancer. Participants also received a CD or MP3 with 20 min of breathing and relaxation exercises to practice at home daily for at least 5 min.	83 women with breast cancer (stage I–III).	Fatigue: Multidimensional Fatigue Inventory (MFI); Fatigue Quality List (FQL); 30-item quality of life questionnaire—C of the European Organization for Research and Treatment of Cancer (EORTC-QLQ-C-30).	**Fatigue** (ANCOVA p, ƞ^2^) T1 = 0.513 (0.007). T2 = 0.664 (0.003). **Nausea and vomiting** between-group intervention effects (ANCOVA p, ƞ^2^). T1 = 0.807 (0.001). T2 = 0.004 (0.122). **Diarrhea** between-group intervention effects (ANCOVA p, ƞ^2^). T1 = 0.446 (0.009). T2 = 0.968 (0.000). **Constipation** between-group intervention effects (ANCOVA p, ƞ^2^). T1 = 0.431 (0.009). T2 = 0.577 (0.005).
[49]	Sensorimotor exercise.	Sensorimotor exercises, which were given twice per week during chemotherapy and up to 6 weeks after it ended.	36 women with breast cancer.	Multidimensional Fatigue Inventory (MFI-20).	**Fatigue** Absence of significant improvements in psychological parameters, as indicated by the MFI scale.
[50]	Walking exercise.	A 30-min walking program in two 5-day periods, without breaks and at a specific time each day, in the following phases: warm-up and body preparation (5 min), brisk walking according to one’s tolerance (10 min), slow walking and body cooling (5 min), followed by 10 min rest and relaxation. The severity of the walking exercise program was mild to moderate and was based on 40-60% of the maximum heart rate.	50 patients with acute myeloid leukemia.	Brief Fatigue Inventory (BFI).	The mean intensity of CRF (**Cancer-Related Fatigue**) in the 24 h preceding the program was compared with the intensity measured on the fifth and tenth days after the intervention. The results showed a significant reduction in CRF on both the fifth and tenth days post-intervention (*p* < 0.001).
[51]	Baduanjin Qigong Exercise.	A series of aerobic exercises believed to impart a silky quality to the body and its energy and to improve general health. It can be broken down into eight exercises that focus on different physical areas and meridians.	90 patients with colorectal cancer (stage I–III).	Brief Fatigue Inventory (BFI).	At 24 weeks, the incidence of moderate-to-severe **fatigue** was significantly reduced in the exercise group compared to the routine care group (23.2% vs. 59.1%, *p* < 0.01).
[52]	Guided Imagery	The patients in the intervention group listened to an audio file for 20 min every day during the interval between two chemotherapy sessions. The file included effective sentences with a calming background.	52 cancer patients in the first course of chemotherapy.	Symptom Distress Scale (SDS).	**Side effects**: independent *t*-test (between groups) post-intervention = −8.47 (*p* = 0.001).
[53]	Nursing intervention.	Standard oncology care plus proactive telephone nursing guidance and support during the first 2 cycles of chemotherapy administration. Each intervention participant was scheduled to receive two scheduled nurse practitioner calls following the first chemotherapy administration and two scheduled calls following the second chemotherapy administration.	120 patients with nonmetastatic (stage I-III) breast (BC), colorectal (CRC), or lung cancer (LC).	Memorial Symptom Assessment Scale-Short Form (MSAS-SF).	None of the outcomes differed between the randomized groups: Number of symptoms (*p* = 0.80); Symptom distress (*p* = 0.86).
[54]	Physical activity.	150 min of moderate to intense physical activity.	85 cancer patients.	Piper Fatigue Scale (PFS); The European Organization for Research and Treatment of Cancer Quality of Life Questionnaire C30 (EORTC-QLQ-C-30).	**Fatigue** Physically active vs. Physically inactive: 21.63 (8.79) vs. 46.85 (5.03) (*p* = 0.01) (R^2^ = 0.11). **Pain** Physically active vs. Physically inactive: 31.60 (8.01) vs. 54.42 (4.58) (*p* = 0.01) (R^2^ = 0.25). **Diarrhea** Physically active vs. Physically inactive: 4.62 (6.98) vs. 21.31 (3.99) (*p* = 0.04) (R^2^ = 0.02). **Constipation** Physically active vs. Physically inactive: 17.28 (6.74) vs. 17.09 (3.88) (*p* = 0.98) (R^2^ = 0.03).
[55]	Physical exercise.	The six-month intervention begins at the start of chemotherapy and includes supervised group resistance training twice weekly at public gyms. Patients also engage in home-based high-intensity interval training twice weekly and low- to moderate-intensity endurance training with 150 min per week of walking or cycling. For breast cancer patients undergoing neoadjuvant treatment, the exercise component lasts four months before surgery. The intervention was developed with input from clinicians, patient representatives, and experts in exercise physiology and physiotherapy.	577 cancer patients (excluded: patients with stage III B-IV cancer).	Multidimensional Fatigue Inventory (MFI) measuring general, physical and mental fatigue, reduced motivation and reduced activity	Participants randomized to exercise at high compared with low-to- moderate intensity had lower MFI **physical fatigue** (adjusted mean difference −1.05 [95% CI, −1.85 to −0.25]) scales.
[56]	Auricular acupressure.	Five ear acupoints (lung, Shen Men, subcortex, liver, and spleen) were selected for the acupressure intervention, which used Semen Vaccariae (SV) and magnetic beads. The procedure involved cleaning the skin with alcohol, applying SV on tape to each acupoint, and pressing until the patient felt swelling pain. Each acupoint was pressed for 20–30 s, 4–6 times per session, with sessions held five times daily (morning, after meals, and before bedtime). The SV tape was replaced every three days. The intervention spanned three chemotherapy cycles, with one cycle consisting of six replacements, and subsequent cycles separated by 3 days. Patients in Groups A and B were trained to self-administer acupressure.	100 cancer patients.	Cancer Fatigue Scale Chinese version (CFS-C).	**Fatigue** Semen Vaccariae vs. Routine Care: −3.88 (−4.79 to 2.96). Magnetic Bead vs. Routine Care: −2.12 (−3.04 to 1.20). Semen Vaccariae vs. Magnetic Bead: −1.75 (−2.69 to 0.82). *p* < 0.01
[57]	Viewing Disney movies.	Participants either watched Disney movies or did not during six cycles of chemotherapy. Films selected were produced between 1950 and 1989 to evoke nostalgia and featured slower plots, avoiding exceptionally depressing content. Each film featured strong, moral protagonists and happy endings. Patients viewed eight films in total, each lasting between 76 and 140 min, during their chemotherapy sessions: approximately 4 h for carboplatin and paclitaxel, and 90 min for carboplatin and pegylated liposomal doxorubicin. Patients chose the movies and used headphones to manage audio levels, minimizing distractions.	55 women with gynecological cancer.	The European Organization for Research and Treatment of Cancer Quality of Life Questionnaire FA12 (EORTC QLQ-FA12).	**Fatigue** Mean [SD] fatigue scores: 85.5 [13.6] vs. 66.4 [22.5]; maximum test (*p* = 0.01).
[58]	Progressive muscle relaxation and early health intervention program.	Participants in the control group received standard chemotherapy care. In contrast, the experimental group received an additional 40-min intervention, including education on managing chemotherapy side effects and a demonstration of the progressive muscle relaxation (PMR) technique. Educational materials and follow-up phone calls supported ongoing PMR practice during treatment, to be done two times daily till the end of chemotherapy.	340 women with breast cancer (stage I–III).	The Common Terminology Criteria for Adverse Events (CTCAE) version 3.0.	**Fatigue** Z# (Mann-Whitney U test) = 13.42 (*p* < 0.01). **Pain** Z# (Mann-Whitney U test) = 1.52 (*p* = 0.128). **Nausea** Z# (Mann-Whitney U test) = 12.56 (*p* < 0.01). **Vomiting** Z# (Mann-Whitney U test) = 12.59 (*p* < 0.01). **Diarrhea** Z# (Mann-Whitney U test) = 1.01 (*p* = 0.311). **Constipation** Z# (Mann-Whitney U test) = 3.12 (*p* = 0.002).
[59]	Laughter yoga.	The intervention group participated in four laughter yoga sessions, each lasting 20–30 min, with sessions held weekly. Each session included 15 laughter yoga steps, with each laugh lasting 30 to 45 s. Sessions were led by trained researchers and supervised to ensure proper technique. The laughter yoga was performed in standing positions and conducted before chemotherapy. In contrast, the control group received routine self-care training, consisting of face-to-face education and pamphlets, delivered weekly for 10 min over four weeks.	69 patients with non-metastatic cancer (excluded: metastatic cancer, upper gastrointestinal cancer).	European Organization for the Research and Treatment of Cancer Quality of Life Questionnaire version 3 (EORTC QLQ-C30).	**Fatigue**: pre- and post- intervention difference. Intervention group (Mean ± SD): −8.82 ± 22.01. Control group (Mean ± SD): −0.95 ± 7.31. *p* = 0.001 **Pain**: pre- and post- intervention difference. Intervention group (Mean ± SD): −8.33 ± 11.78. Control group (Mean ± SD): 0.95 ± 11.39. *p* = 0.001 **Diarrhea**: pre- and post- intervention difference. Intervention group (Mean ± SD): −1.96 ± 7.96. Control group (Mean ± SD): −0.95 ± 9.58. *p* = 0.650 **Constipation**: pre- and post- intervention difference. Intervention group (Mean ± SD): −0.98 ± 10.00. Control group (Mean ± SD): 0.95 ± 5.63. *p* = 0.321
[60]	Network-based positive psychological nursing intervention.	Participants were divided into two groups: a control group receiving traditional treatment, and a study group that received routine nursing care along with web-based psychological support. Routine care included patient education, advice on managing chemotherapy side effects, and family involvement. The study group also received continuous mobile-based communication, online educational resources, and regular psychological counseling. Additionally, they participated in group interventions to boost self-esteem and received lifestyle counseling focused on nutrition, exercise, and sleep. Before discharge, they were given detailed guidance on managing complications and follow-up care.	101 patients with cervical cancer.	Revised Piper Fatigue Scale (RPFS).	**Fatigue**: Total score before intervention vs. after intervention (*x* ± s) Study group: 28.49 *±* 3.12 vs. 19.01 *±* 2.11 (*p* < 0.001). Control group: 28.16 *±* 3.08 vs. 21.94 *±* 2.18 (*p* = 0.601).
[61]	Progressive muscle relaxation training.	The therapist directed the patient which group of muscles to tense and release each time and how long to tense them for (usually a few seconds). Tension–release of groups of muscles was followed by deep breathing. Administration of such a session took about 25 min, and each session was followed by a few minutes of guided imagery.	71 Chinese chemotherapy-naive breast cancer patients.	Morrow Assessment of Nausea and Vomiting (MANE).	**Nausea** Frequency: experimental group vs. control group the first 4 days after chemotherapy (*p* < 0.05) Duration (min) (experimental vs. control) (*p* < 0.05). Day 1: 59.5 vs. 82.1. Day 2: 136.6 vs. 276.5. Day 7: 0.74 vs. 2.3. Intensity (*p* > 0.05). **Vomiting** Frequency: experimental group vs. control group the first 4 days after chemotherapy (*p* < 0.05). Duration (min) (experimental vs. control) (*p* = 0.016). Day 1: 8 vs. 40.3. No reported vomiting episodes by day 8 after chemotherapy for both groups.
[62]	Cognitive behavioral intervention.	A cognitive-behavioral intervention aimed at helping patients acquire skills and strategies for effective self-management of their symptoms. The process involves the patient and nurse identifying issues, with the nurse proposing solutions and assessing the patient’s readiness to apply cognitive and behavioral techniques. Patients’ symptoms were evaluated during intake and at the 10- and 20-week assessments.	237 cancer patients.	15-item symptom severity index.	By the end of the intervention, the percentage of patients who had implemented strategies for lowering severity below baseline threshold for selected symptoms were **constipation** (70%), **fatigue** (46%), **nausea** (56%), **pain** (50%), and **diarrhea** (72%).
[63]	Progressive muscle relaxation training (PMRT) and guided imagery (GI).	Guided imagery was initiated after the third session of PMRT, during which patients were taught to imagine a peaceful scene of their own choice to enhance relaxation. After PMRT, the patient was guided in the use of visual imagery to achieve a deeper state of relaxation. The procedures and methods from the third to the sixth session were identical.	60 cancer patients (excluded: patients with stage IV breast cancer and life expectancy under 6 months).	Self-reported (7-point, one-item, Likert scale method).	**Nausea** Similar between groups in the first four sessions, but the PMRT and GI group had significantly less nausea by the 5th and 6th sessions. Group effect: F = 4.16 (*p* < 0.05). Group x session effect: F = 2.78 (*p* < 0.05). **Vomiting** Peaked in the PMRT and GI group during the 1st and 3rd sessions. Group x session effect: F = 2.56 (*p* < 0.05).
[64]	Massage.	Effleurage massage– a form of massage involving a repeated circular stroking movement made with the palm of the hand. While chemotherapy was being administered, a massage was given in the chemotherapy ward for 20 min. Five massage therapy sessions were provided in total.	39 women with breast cancer (Stage I-II A-II B-III).	Visual Analogue Scale (VAS).	**Nausea** Mean improvement: 73.2% (SD 32.3) (median/interquartile range 80%/40–100) (*p* = 0.025).
[65]	Group psychotherapy.	Group discussions addressed various aspects of cancer treatment and its side effects. The format included a 30-min presentation by an oncology resident and a social worker, followed by detailed information for another 30 min. The final hour was dedicated to an open discussion where new and experienced patients, along with their families, could ask questions and share experiences. Family members of Group A were also encouraged to participate.	100 patients treated with chemotherapy for breast and lung cancer (stage III B, IV).	Common Terminology Criteria for Adverse Events (CTCAE).	**Vomiting** Grade 2 and above: experimental group (6.2 ± 2.7) vs. control group (13.4 ± 3.8) (*p* < 0.05).
[66]	Integrated yoga program.	The yoga intervention involved asanas, breathing exercises, pranayama, meditation, and imagery-based relaxation techniques. Participants practiced yoga for 30 min before each chemotherapy infusion and were assigned daily homework, including one hour of practice six days a week, with a minimum of three hours per week but ideally six hours of home practice weekly.	62 women with breast cancer.	Morrow Assessment of Nausea and Emesis (MANE).	**Nausea** Post-chemotherapy frequency: experimental group (3.6 ± 1.6) vs. control group (4.5 ± 0.9). *t*-value (d.f.) = −2.67 (60) (*p* = 0.01). Post-chemotherapy intensity: experimental group (2.3 ± 1.2) vs. control group (3.4 ± 1.1). *t*-value (d.f.) = −3.71 (57) (*p* < 0.001). Anticipatory frequency: experimental group (1.3 ± 0.98) vs. control group (1.9 ± 1.3). *t*-value (d.f.) = −1.90 (60) (*p* = 0.06). Anticipatory intensity: experimental group (0.6 ± 1.03) vs. control group (1.7 ± 1.5). *t*-value (d.f.) = −3.17 (55) (*p* = 0.003). **Vomiting** Post-chemotherapy frequency: experimental group (2.3 ± 1.4) vs. control group (2.9 ± 1.4). *t*-value (d.f.) = −1.90 (58) (*p* = 0.06). Post-chemotherapy intensity: experimental group (1.6 ± 1.0) vs. control group (2.2 ± 1.4). *t*-value (d.f.) = −1.99 (60) (*p* = 0.05). Anticipatory frequency: experimental group (1.1 ± 0.88) vs. control group (1.2 ± 0.73). *t*-value (d.f.) = −0.476 (53) (*p* = 0.63) Anticipatory intensity: experimental group (0.3 ± 0.67) vs. control group (0.87 ± 1.3). *t*-value (d.f.) = −2.05 (56) (*p* = 0.04).
[67]	Guided visual imagery and music therapy.	Patients chose one of five nature paintings for guided visual imagery, each accompanied by a unique CD of soft, serene Turkish instrumental music. They were instructed to imagine themselves in the scene depicted by their chosen painting and to play the corresponding music 15 min before their chemotherapy session.	40 cancer patients.	Morrow Assessment of Nausea and Emesis (MANE); Visual Analogue Scale (VAS).	**Nausea** Pre-Chemotherapy Score 2.28 ± 2.532 in the third cycle (significant decrease, *p* < 0.05).
[68]	Acupressure.	Patients in the acupressure group wore bracelets with a 1 cm plastic button positioned at the P6 acupoint, located three finger widths above the wrist crease on the forearm. They were instructed to start wearing the bracelets the morning before chemotherapy and continue for the following seven days. The sham group received similar wristbands with the button placed on the outside, and they were instructed to wear them with the button away from the P6 point, along with standard antiemetic treatment.	500 cancer patients.	The Rhodes Index of Nausea, Vomiting and Retching (Rhodes Index); The MASCC Antiemesis Tool (MAT).	The trial found no statistically significant differences in **nausea and vomiting** between the three groups. However, both wristband groups showed a higher likelihood of improved nausea compared to the standard care group, with the sham wristband group performing better than the acupressure group. Females in the wristband groups experienced significantly greater improvements compared to males.
[69]	Guided Imagery.	Patients listened to two 10-min audio-recorded guided imagery tracks on separate CDs. The first track featured soothing nature sounds, while the second included imagery designed to improve feelings. Patients were instructed to listen to the first track the night before the third session.	55 Iranian breast cancer (stage I, II or III) patients.	Morrow Assessment of Nausea and Emesis (MANE).	**Nausea**: Pre-Chemotherapy Severity: Decreased from 1.91 ± 1.97 in the second cycle to 1.28 ± 0.85 in the third cycle. Post-Chemotherapy Severity: Decreased from 2.07 ± 1.63 in the second cycle to 0.98 ± 0.84 in the third cycle. Pre-Chemotherapy Severity: Decreased from 0.48 ± 0.09 in the second cycle to 0 ± 0 in the third cycle (*p* < 0.05). Post-Chemotherapy Severity: Decreased from 0.62 ± 0.05 in the second cycle to 0 ± 0 after chemotherapy. *p* = 0.0001
[70]	Yoga.	Participants in the study group started practicing yoga and pranayama two days before their scheduled chemotherapy and continued throughout the chemotherapy cycle and the days following treatment.	100 cancer patients.	Ad hoc scales.	**Nausea** Insignificant reduction in the incidence (90% vs. 78%, *p* = 0.35). **Vomiting** Significant reduction in the incidence (42% vs. 22%, *p* = 0.01).
[71]	Nurse-administered behavioral interventions	A low-intensity intervention involved listening to soothing music and receiving standard care, while a high-intensity intervention included relaxation and meditation techniques. The high-intensity intervention, called mindful relaxation, featured a script combining guided imagery, mindfulness meditation, and yoga, tailored for medically unwell patients. The low-intensity intervention involved listening to relaxing music for a similar duration.	474 patients undergoing chemotherapy for solid tumors.	Morrow Assessment of Nausea and Emesis (MANE).	**Nausea**: Severity at the midpoint: Chi-square = 12.7. (*p* = 0.01) **Vomiting**: The prevalence of vomiting was low and the difference between treatment groups was not significant.
[72]	Foot reflexology.	Patients in the foot reflexology group received four 30-min sessions during chemotherapy, spaced every 2 or 3 weeks. The reflexologist provided instructions on stimulating specific hand reflexology zones to address nausea, focusing on upper and lower digestive reflex points and areas related to smooth muscle metabolism, including the lymphatic system, kidneys, bladder, lungs, thyroid, and parathyroid.	80 patients with lung or digestive cancer diagnosis (stage IV, III B, III A, II).	Visual Analogue Scale (VAS).	**Nausea** Delayed n (%) experimental group vs. control group. Cycle 2: 11 (50) vs. 18 (62). Cycle 3: 9 (43) vs. 17 (61). Cycle 4: 7 (35) vs. 15 (58). End: 7 (35) vs. 12 (48) (*p* = 0.28). **Vomiting** Delayed n (%) experimental group vs. control group. Cycle 2: 5 (23) vs. 5 (17). Cycle 3: 3 (14) vs. 5 (18). Cycle 4: 4 (20) vs. 4 (15). End: 4 (20) vs. 4 (16) (*p* = 0.99). **Antiemetic drug use** n (%) experimental group vs. control group. Cycle 2: 5 (23) vs. 12 (41). Cycle 3: 2 (10) vs. 11 (39). Cycle 4: 3 (15) vs. 10 (38). End: 2 (10) vs. 7 (28) (*p* = 0.04).
[73]	Virtual Reality (VR)	Participants in the intervention group received a single session of immersive virtual reality (VR) during their chemotherapy treatment, while the control group received standard nursing care. The intervention used VR headsets with visual and audio features, allowing participants to explore various virtual environments such as rivers, forests, and mountains. The oncology nursing staff provided instructions on using the devices and ensured comfort.	100 patients diagnosed with any stage of lung cancer (Easter Cooperative Oncology Group Performance Status ≤ 2).	Edmonton Symptom Assessment Scale (ESAS).	**Pain**. VR group vs control: mean (SD). Pre-chemotherapy: 1.60 (2.705) vs. 1.82 (2.847) (*p* = 0.69). Post-chemotherapy: 1.11 (1.948) vs. 1.67 (2.900) (*p* = 0.26). **Nausea**. VR group vs control: mean (SD). Pre-chemotherapy: 0.93 (2.053) vs. 0.62 (1.585) (*p* = 0.42). Post-chemotherapy: 0.49 (1.339) vs. 0.76 (1.694) (*p* = 0.39).
[74]	Nursing intervention.	The control group received routine nursing care, while the intervention group followed the Nurse-led Intervention Based on Risk Assessment (NIBRA).	84 breast cancer patients.	Functional living index-emesis (FLIE).	**Nausea level**: intervention *vs* control group [M (P25, P75)]. Acute: 3.00 (0.00, 5.00) vs. 4.00 (2.00, 6.00) (*p* = 0.91). Delayed: 2.00 (0.00, 4.00) vs. 4.00 (2.00, 7.00) (*p* = 0.006). **Vomiting frequency**: intervention vs. control group [M (P25, P75)]. Acute: 1.00 (0.00, 2.25) vs. 2.00 (2.00, 4.00) (*p* = 0.146). Delayed: 3.50 (0.00, 6.00) vs. 5.00 (2.75, 8.00) (*p* = 0.027).
[75]	Empowerment program.	Participants were divided into two groups: one received standard nursing care with advice on managing chemotherapy side effects, while the other received additional support through an educational program and a chatbot. The standard care group got general advice from nurses on side effects, diet, and hygiene. The intervention group was split into two subgroups. One received face-to-face education from nurses on managing side effects, with sessions conducted on the first day of chemotherapy. Data collection for this subgroup was from April to July 2021. The other subgroup used the ChemoFreeBot, a chatbot developed to provide information and answer questions about chemotherapy. Participants interacted with the chatbot via WhatsApp.	150 women with breast cancer (excluded metastatic cancer).	The Memorial Symptoms Assessment Scale (MSAS).	**Nausea and vomiting** **Constipation and diarrhea** **Physical symptoms frequency**: ChemoFreeBot vs. Routine care: *p* < 0.001. ChemoFreeBot vs. Nurse-led education: *p* < 0.001. Routine care vs. Nurse-led education: *p* < 0.001. **Physical symptoms severity**: ChemoFreeBot vs. Routine care: *p* < 0.001. ChemoFreeBot vs. Nurse-led education: *p* < 0.001. Routine care vs. Nurse-led education: *p* < 0.001. **Physical symptoms distress**: ChemoFreeBot vs. Routine care: *p* < 0.001. ChemoFreeBot vs. Nurse-led education: *p* < 0.001. Routine care vs. Nurse-led education: *p* = 1.
[76]	Reiki therapy.	Four Reiki therapy sessions–a gentle touch energy healing practice that promotes relaxation and lowers stress and anxiety.	118 cancer patients.	Visual Analogue Scale (VAS).	**Pain** From 4.44 ± 3.22 to 2.32 ± 2.38 (*p* < 0.0191) for patients who underwent the full cycle of four sessions. Most of the benefits were achieved in the earlier three sessions.
[77]	Multimodal exercise.	Three phases: the first one lasts for 15 min and includes balance training (10 min) and coordination practices (5 min); the second one schedules endurance training (10 min) and resistance training (30 min). The last phase is designed for the cool down, and it lasts 10–15 min.	30 cancer patients with CIPN.	Trial Outcome Index (TOI).	**Pain**: Change between groups: t1-t0: *p* = 0.028 t2-t0: *p* = 0.031 t2-t1: *p* = 0.592

## Data Availability

No new data were created or analyzed in this study. Data sharing is not applicable to this article.

## Appendix A

**Table A1:** Evaluation of the methodological quality according to the NIH checklist.

Article	Poor	Fair	Good
[29]	✓		
[33]		✓	
[34]	✓		
[35]	✓		
[36]	✓		
[37]		✓	
[38]		✓	
[39]		✓	
[40]		✓	
[41]		✓	
[43]		✓	
[43]	✓		
[44]		✓	
[45]	✓		
[46]			✓
[47]	✓		
[48]	✓		
[49]	✓		
[50]		✓	
[51]		✓	
[52]	✓		
[53]		✓	
[54]		✓	
[55]		✓	
[56]		✓	
[57]		✓	
[58]		✓	
[59]		✓	
[60]		✓	
[60]	✓		
[61]	✓		
[64]		✓	
[65]	✓		
[66]		✓	
[67]		✓	
[68]		✓	
[69]		✓	
[70]	✓		
[71]		✓	
[72]	✓		
[72]		✓	
[73]	✓		
[74]	✓		
[75]		✓	
[76]	✓		
[77]		✓	

## References

[1] New Report on Global Cancer Burden in 2022 by World Region and Human Development Level. https://www.iarc.who.int/news-events/new-report-on-global-cancer-burden-in-2022-by-world-region-and-human-development-level/.

[2] Bray F., Laversanne M., Sung H., Ferlay J., Siegel R.L., Soerjomataram I., Jemal A. (2024). Global cancer statistics 2022: GLOBOCAN estimates of incidence and mortality worldwide for 36 cancers in 185 countries. CA Cancer J. Clin..

[3] Siegel R.L., Giaquinto A.N., Jemal A. (2024). Cancer statistics, 2024. CA Cancer J. Clin..

[4] Harley C., Pini S., Bartlett Y.K., Velikova G. (2012). Defining chronic cancer: Patient experiences and self-management needs. BMJ Support. Palliat. Care.

[5] Ke X., Shen L. (2017). Molecular targeted therapy of cancer: The progress and future prospect. Front. Lab. Med..

[6] Zhong L., Li Y., Xiong L., Wang W., Wu M., Yuan T., Yang W., Tian C., Miao Z., Wang T. (2021). Small molecules in targeted cancer therapy: Advances, challenges, and future perspectives. Signal Transduct. Target. Ther..

[7] Pearce A., Haas M., Viney R., Pearson S.-A., Haywood P., Brown C., Ward R. (2017). Incidence and severity of self-reported chemotherapy side effects in routine care: A prospective cohort study. PLoS ONE.

[8] Katta B., Vijayakumar C., Dutta S., Dubashi B., Ramakrishnaiah V.P.N. (2023). The Incidence and Severity of Patient-Reported Side Effects of Chemotherapy in Routine Clinical Care: A Prospective Observational Study. Cureus.

[9] Bower J.E. (2014). Cancer-related fatigue—Mechanisms, risk factors, and treatments. Nat. Rev. Clin. Oncol..

[10] Altun İ., Sonkaya A. (2018). The Most Common Side Effects Experienced by Patients Were Receiving First Cycle of Chemotherapy. Iran. J. Public. Health.

[11] Nurgali K., Jagoe R.T., Abalo R. (2018). Adverse Effects of Cancer Chemotherapy: Anything new to improve tolerance and reduce sequelae?. Front. Pharmacol..

[12] Brianna N., Lee S.H. (2023). Chemotherapy: How to reduce its adverse effects while maintaining the potency?. Med. Oncol..

[13] Azemmour Y., Boutayeb S., Beddaa H., Errihani H. (2022). Physical activity in cancer care: Barriers and interventions. Pan Afr. Med. J..

[14] Jermini M., Fonzo-Christe C., Blondon K., Milaire C., Stirnemann J., Bonnabry P., Guignard B. (2024). Financial impact of medication reviews by clinical pharmacists to reduce in-hospital adverse drug events: A return-on-investment analysis. Int. J. Clin. Pharm..

[15] Di Mattei V.E., Perego G., Taranto P., Mazzetti M., Marotta E., Candiani M., Salvatore S. (2020). The Long-Term Effects of Cancer Treatment on Sexuality and couple relationships. Fam. Process.

[16] Perego G., Di Mattei V.E., Mazzetti M., Milano F., Gatti C., Rancoita P.M.V., Taranto P., Rabaiotti E., Cioffi R., Candiani M. (2023). The experience of COVID-19 in a sample of gynecological cancer patients undergoing chemotherapy: A focus on the psychological implications. Int. J. Environ. Res. Public. Health.

[17] Strimbu K., Tavel J.A. (2010). What are biomarkers?. Curr. Opin. HIV AIDS.

[18] Lotfi-Jam K., Carey M., Jefford M., Schofield P., Charleson C., Aranda S. (2008). Nonpharmacologic Strategies for Managing common Chemotherapy Adverse Effects: A Systematic review. J. Clin. Oncol..

[19] Hökkä M., Kaakinen P., Pölkki T. (2014). A systematic review: Non-pharmacological interventions in treating pain in patients with advanced cancer. J. Adv. Nurs..

[20] Li K., Cai Y., Xie S., Zhou Y., Dong J., Zhu Q., Zhang J., Qiu X. (2022). Evidence Summary for Nonpharmacological Management of Chemotherapy-Induced nausea and vomiting. BioMed Res. Int..

[21] Van Den Boogaard W.M.C., Komninos D.S.J., Vermeij W.P. (2022). Chemotherapy Side-Effects: Not all DNA damage is equal. Cancers.

[22] Page M.J., McKenzie J.E., Bossuyt P.M., Boutron I., Hoffmann T.C., Mulrow C.D., Shamseer L., Tetzlaff J.M., Akl E.A., Brennan S.E. (2021). The PRISMA 2020 statement: An updated guideline for reporting systematic reviews. BMJ.

[23] Wolsko P.M., Eisenberg D.M., Davis R.B., Phillips R.S. (2004). Use of mind-body medical therapies. J. Gen. Intern. Med..

[24] Sanadgol S., Firouzkouhi M., Badakhsh M., Abdollahimohammad A., Shahraki-Vahed A. (2020). Effect of guided imagery training on death anxiety of nurses at COVID-19 intensive care units: A quasi-experimental study. Neuropsychiatr. I Neuropsychol..

[25] Zargarzadeh M., Shirazi M. (2014). The effect of progressive muscle relaxation method on test anxiety in nursing students. Iran. J. Nurs. Midwifery Res..

[26] Keptner K.M., Fitzgibbon C., O’Sullivan J. (2020). Effectiveness of anxiety reduction interventions on test anxiety: A comparison of four techniques incorporating sensory modulation. Br. J. Occup. Ther..

[27] Sengupta P. (2012). Health Impacts of Yoga and Pranayama: A State-of-the-Art Review. Int. J. Prev. Medicine.

[28] Bennett S., Pigott A., Beller E.M., Haines T., Meredith P., Delaney C. (2016). Educational Interventions for the Management of Cancer-Related Fatigue in Adults.

[29] Oldenmenger W., Sillevis Smitt P., Montfort K., de Raaf P., van der Rijt C. (2011). A combined pain consultation and pain education program decreases average and current pain and decreases interference in daily life by pain in oncology outpatients: A randomized controlled trial. Pain.

[30] Penedo F.J., Benedict C., McGregor B. (2013). Cancer: Psychosocial Treatment. Encyclopedia of Behavioral Medicine.

[31] Tabish S.A. (2008). Complementary and Alternative Healthcare: Is it Evidence-based?. Int. J. Health Sci..

[32] Adams M., Jewell A.P. (2007). The use of complementary and alternative medicine by cancer patients. Int. Semin. Surg. Oncol..

[33] Mock V., Frangakis C., Davidson N.E., Ropka M.E., Pickett M., Poniatowski B., Stewart K.J., Cameron L., Zawacki K., Podewils L.J. (2005). Exercise manages fatigue during breast cancer treatment: A randomized controlled trial. Psycho-oncology.

[34] Andersen C., Adamsen L., Moeller T., Midtgaard J., Quist M., Tveteraas A., Rorth M. (2006). The effect of a multidimensional exercise programme on symptoms and side-effects in cancer patients undergoing chemotherapy—The use of semi-structured diaries. Eur. J. Oncol. Nurs..

[35] Armes J., Chalder T., Addington-Hall J., Richardson A., Hotopf M. (2007). A randomized controlled trial to evaluate the effectiveness of a brief, behaviorally oriented intervention for cancer-related fatigue. Cancer.

[36] Chang P.-H., Lai Y.-H., Shun S.-C., Lin L.-Y., Chen M.-L., Yang Y., Tsai J.-C., Huang G.-S., Cheng S.-Y. (2008). Effects of a walking Intervention on Fatigue-Related Experiences of Hospitalized Acute myelogenous leukemia patients undergoing chemotherapy: A randomized controlled trial. J. Pain Symptom Manag..

[37] Kearney N., Miller M., Maguire R., Dolan S., MacDonald R., McLeod J., Maher L., Sinclair L., Norrie J., Wengström Y. (2008). WISECARE+: Results of a European study of a nursing intervention for the management of chemotherapy-related symptoms. Eur. J. Oncol. Nurs..

[38] Berger A.M., Kuhn B.R., Farr L.A., Lynch J.C., Agrawal S., Chamberlain J., Von Essen S.G. (2008). Behavioral therapy intervention trial to improve sleep quality and cancer-related fatigue. Psycho-oncology.

[39] Barsevick A., Beck S.L., Dudley W.N., Wong B., Berger A.M., Whitmer K., Newhall T., Brown S., Stewart K. (2010). Efficacy of an intervention for fatigue and sleep disturbance during cancer chemotherapy. J. Pain Symptom Manag..

[40] Yang C.-Y., Tsai J.-C., Huang Y.-C., Lin C.-C. (2010). Effects of a home-based walking program on perceived symptom and mood status in postoperative breast cancer women receiving adjuvant chemotherapy. J. Adv. Nurs..

[41] Lin K.-Y., Shun S.-C., Lai Y.-H., Liang J.-T., Tsauo J.-Y. (2014). Comparison of the effects of a supervised exercise program and usual care in patients with colorectal cancer undergoing chemotherapy. Cancer Nurs..

[42] Charalambous A., Giannakopoulou M., Bozas E., Marcou Y., Kitsios P., Paikousis L. (2016). Guided imagery and progressive muscle relaxation as a cluster of symptoms management intervention in patients receiving chemotherapy: A randomized control trial. PLoS ONE.

[43] Şahin Z.A., Ergüney S. (2015). Effect on symptom management education receiving patients of chemotherapy. J. Cancer Educ..

[44] Chuang T.-Y., Yeh M.-L., Chung Y.-C. (2017). A nurse facilitated mind-body interactive exercise (Chan-Chuang qigong) improves the health status of non-Hodgkin lymphoma patients receiving chemotherapy: Randomised controlled trial. Int. J. Nurs. Stud..

[45] Da Silva Alves R., Iunes D.H., Pereira I.C., Borges J.B.C., Nogueira D.A., Silva A.M., Lobato D.F.M., Carvalho L.C. (2017). Influence of exergaming on the perception of Cancer-Related Fatigue. Games Health J..

[46] Zhang B., Dong J.-N., Sun P., Feng C., Liu Y.-C. (2017). Effect of therapeutic care for treating fatigue in patients with breast cancer receiving chemotherapy. Medicine.

[47] Coolbrandt A., Wildiers H., Laenen A., Aertgeerts B., De Casterlé B.D., Van Achterberg T., Milisen K. (2018). A nursing intervention for reducing symptom burden during chemotherapy. Oncol. Nurs. Forum.

[48] Jong M.C., Boers I., Van Der Velden A.P.S., Van Der Meij S., Göker E., Timmer-Bonte A.N.J.H., Van Wietmarschen H.A. (2018). A Randomized Study of Yoga for Fatigue and Quality of Life in Women with Breast Cancer Undergoing (Neo) Adjuvant Chemotherapy. J. Altern. Complement. Med..

[49] Vollmers P.L., Mundhenke C., Maass N., Bauerschlag D., Kratzenstein S., Röcken C., Schmidt T. (2018). Evaluation of the effects of sensorimotor exercise on physical and psychological parameters in breast cancer patients undergoing neurotoxic chemotherapy. J. Cancer Res. Clin. Oncol..

[50] Gheyasi F., Baraz S., Malehi A., Ahmadzadeh A., Salehi R., Vaismoradi M. (2019). Effect of the Walking Exercise Program on Cancer-Related Fatigue in Patients with Acute Myeloid Leukemia Undergoing Chemotherapy. Asian Pac. J. Cancer Prev..

[51] Lu Y., Qu H.-Q., Chen F.-Y., Li X.-T., Cai L., Chen S., Sun Y.-Y. (2019). Effect of Baduanjin Qigong Exercise on Cancer-Related Fatigue in Patients with Colorectal Cancer Undergoing Chemotherapy: A Randomized Controlled Trial. Oncol. Res. Treat..

[52] Mahdizadeh M.J., Tirgari B., Abadi O.S.R.R., Bahaadinbeigy K. (2019). Guided imagery: Reducing anxiety, depression, and selected side effects associated with chemotherapy. Clin. J. Oncol. Nurs..

[53] Traeger L., McDonnell T.M., McCarty C.E., Greer J.A., El-Jawahri A., Temel J.S. (2015). Nursing intervention to enhance outpatient chemotherapy symptom management: Patient-reported outcomes of a randomized controlled trial. Cancer.

[54] Caetano A.F.P., Silva D.A.S., Martins P.C., De Oliveira Toscano J.J. (2020). Impact of physical activity on fatigue and quality of life of cancer patients. Rev. Bras. De Med. Do Esporte.

[55] Demmelmaier I., Brooke H.L., Henriksson A., Mazzoni A., Bjørke A.C.H., Igelström H., Ax A., Sjövall K., Hellbom M., Pingel R. (2021). Does exercise intensity matter for fatigue during (neo-)adjuvant cancer treatment? The Phys-Can randomized clinical trial. Scand. J. Med. Sci. Sports.

[56] Lin L., Zhang Y., Qian H.Y., Xu J.L., Xie C.Y., Dong B., Tian L. (2019). Auricular acupressure for cancer-related fatigue during lung cancer chemotherapy: A randomised trial. BMJ Support. Palliat. Care.

[57] Pils S., Ott J., Reinthaller A., Steiner E., Springer S., Ristl R. (2020). Effect of viewing Disney Movies during chemotherapy on Self-Reported Quality of Life among Patients with Gynecologic Cancer. JAMA Netw. Open.

[58] Koshy B., Avudaiappan S., Anand A.S. (2024). Efficacy of Early Health Intervention Programs on Adverse Effects of Chemotherapy Among Women With Breast Cancer: A Randomized Controlled Trial. Cureus.

[59] Namazinia M., Mazlum S.R., Mohajer S., Lopez V. (2023). Effects of laughter yoga on health-related quality of life in cancer patients undergoing chemotherapy: A randomized clinical trial. BMC Complement. Med. Ther..

[60] Nie X. (2024). Effects of Network-based Positive Psychological Nursing Model on Negative Emotions, Cancer-related Fatigue, and Quality of Life in Cervical Cancer Patients with Post-operative Chemotherapy. Ann. Ital. Di Chir..

[61] Molassiotis A., Yung H.P., Yam B.M., Chan F.Y., Mok T. (2002). The effectiveness of progressive muscle relaxation training in managing chemotherapy-induced nausea and vomiting in Chinese breast cancer patients: A randomised controlled trial. Support. Care Cancer.

[62] Given C., Given B., Rahbar M., Jeon S., McCorkle R., Cimprich B., Galecki A., Kozachik S., Brady A., Fisher-Malloy M.J. (2004). Effect of a cognitive behavioral intervention on reducing symptom severity during chemotherapy. J. Clin. Oncol..

[63] Yoo H.J., Ahn S.H., Kim S.B., Kim W.K., Han O.S. (2005). Efficacy of progressive muscle relaxation training and guided imagery in reducing chemotherapy side effects in patients with breast cancer and in improving their quality of life. Support. Care Cancer.

[64] Billhult A., Bergbom I., Stener-Victorin E. (2007). Massage Relieves Nausea in Women with Breast Cancer Who Are Undergoing Chemotherapy. J. Altern. Complement. Med..

[65] Parvez T., Alharbi T.M., Mein F.D. (2007). Impact of group psychotherapy in chemotherapy induced vomiting for treatment of advanced breast and lungs cancer. J. Coll. Physicians Surg. Pak..

[66] Raghavendra R.M., Nagarathna R., Nagendra H.R., Gopinath K.S., Srinath B.S., Ravi B.D., Patil S., Ramesh B.S., Nalini R. (2007). Effects of an integrated yoga programme on chemotherapy-induced nausea and emesis in breast cancer patients. Eur. J. Cancer Care.

[67] Karagozoglu S., Tekyasar F., Yilmaz F.A. (2012). Effects of music therapy and guided visual imagery on chemotherapy-induced anxiety and nausea–vomiting. J. Clin. Nurs..

[68] Molassiotis A., Russell W., Hughes J., Breckons M., Lloyd-Williams M., Richardson J., Hulme C., Brearley S.G., Campbell M., Garrow A. (2014). The Effectiveness of acupressure for the control and management of Chemotherapy-Related Acute and Delayed nausea: A randomized controlled trial. J. Pain Symptom Manag..

[69] Hosseini M., Tirgari B., Forouzi M.A., Jahani Y. (2016). Guided imagery effects on chemotherapy induced nausea and vomiting in Iranian breast cancer patients. Complement. Ther. Clin. Pract..

[70] Kothari T.O., Jakhar S.L., Bothra D., Sharma N., Kumar H.S., Baradia M.R. (2019). Prospective randomized trial of standard antiemetic therapy with yoga versus standard antiemetic therapy alone for highly emetogenic chemotherapy-induced nausea and vomiting in South Asian population. J. Cancer Res. Ther..

[71] Hunter J.J., Maunder R.G., Sui D., Esplen M.J., Chaoul A., Fisch M.J., Bassett R.L., Harden-Harrison M.M., Lagrone L., Wong L. (2020). A randomized trial of nurse-administered behavioral interventions to manage anticipatory nausea and vomiting in chemotherapy. Cancer Med..

[72] Murat-Ringot A., Souquet P.J., Subtil F., Boutitie F., Preau M., Piriou V. (2021). The effect of foot reflexology on Chemotherapy-Induced nausea and vomiting in patients with digestive or lung cancer: Randomized controlled trial. JMIR Cancer.

[73] Mitello L., Marti F., Mauro L., Siano L., Pucci A., Tarantino C., Rocco G., Stievano A., Iacorossi L., Anastasi G. (2024). The Usefulness of Virtual Reality in Symptom Management during Chemotherapy in Lung Cancer Patients: A Quasi-Experimental Study. J. Clin. Med..

[74] Ning Y., Zhang L., Zhi X., Zhao Y., Fang Y., Wu B., Xu Z., Huang L., Pei Y. (2024). Application of nurse-led CINV management scheme based on risk assessment in breast cancer patients. Precis. Med. Sci..

[75] Tawfik E., Ghallab E., Moustafa A. (2023). A nurse versus a chatbot—The effect of an empowerment program on chemotherapy-related side effects and the self-care behaviors of women living with breast Cancer: A randomized controlled trial. BMC Nurs..

[76] Birocco N., Guillame C., Storto S., Ritorto G., Catino C., Gir N., Balestra L., Tealdi G., Orecchia C., De Vito G. (2011). The effects of reiki therapy on pain and anxiety in patients attending a day oncology and infusion services unit. Am. J. Hosp. Palliat. Med..

[77] Zimmer P., Trebing S., Timmers-Trebing U., Schenk A., Paust R., Bloch W., Rudolph R., Streckmann F., Baumann F.T. (2017). Eight-week, multimodal exercise counteracts a progress of chemotherapy-induced peripheral neuropathy and improves balance and strength in metastasized colorectal cancer patients: A randomized controlled trial. Support. Care Cancer.

[78] Kumari D., Patil J. (2023). Guided imagery for anxiety disorder: Therapeutic efficacy and changes in quality of life. Ind. Psychiatry J..

[79] Hernandez R., Bassett S.M., Boughton S.W., Schuette S.A., Shiu E.W., Moskowitz J.T. (2017). Psychological Well-Being and Physical Health: Associations, mechanisms, and future directions. Emot. Rev..

[80] Gaston-Johansson F., Fall-Dickson J.M., Nanda J., Ohly K.V., Stillman S., Krumm S., Kennedy M.J. (2000). The effectiveness of the Comprehensive Coping Strategy Program on Clinical Outcomes in Breast Cancer autologous bone marrow transplantation. Cancer Nurs..

[81] Woodyard C. (2011). Exploring the therapeutic effects of yoga and its ability to increase quality of life. Int. J. Yoga.

[82] Williams S.A., Schreier A.M. (2005). The role of education in managing fatigue, anxiety, and sleep disorders in women undergoing chemotherapy for breast cancer. Appl. Nurs. Res..

[83] Mandolesi L., Polverino A., Montuori S., Foti F., Ferraioli G., Sorrentino P., Sorrentino G. (2018). Effects of physical exercise on cognitive functioning and wellbeing: Biological and psychological benefits. Front. Psychol..

[84] McFarland D.C., Shaffer K.M., Tiersten A., Holland J. (2018). Physical symptom burden and its association with distress, anxiety, and depression in breast cancer. Psychosomatics.

[85] Di Mattei V.E., Perego G., Taranto P., Mazzetti M., Rancoita P.M.V., Milano F., Mangili G., Rabaiotti E., Bergamini A., Cioffi R. (2022). Socio-demographic and psychological factors associated with quality of life of women undergoing chemotherapy treatment for gynecological cancer. Support. Care Cancer.

[86] Luszczynska A., Pawlowska I., Cieslak R., Knoll N., Scholz U. (2012). Social support and quality of life among lung cancer patients: A systematic review. Psycho-oncology.

[87] Leszcz M. (2020). Group therapy for patients with medical illness. Am. J. Psychother..

[88] Montross-Thomas L.P., Meier E.A., Reynolds-Norolahi K., Raskin E.E., Slater D., Mills P.J., MacElhern L., Kallenberg G. (2017). Inpatients’ preferences, beliefs, and stated willingness to pay for complementary and alternative medicine treatments. J. Altern. Complement. Med..

[89] Mukhtar K., Nawaz H., Abid S. (2019). Functional gastrointestinal disorders and gut-brain axis: What does the future hold?. World J. Gastroenterol..

[90] Madison A., Kiecolt-Glaser J.K. (2019). Stress, depression, diet, and the gut microbiota: Human–bacteria interactions at the core of psychoneuroimmunology and nutrition. Curr. Opin. Behav. Sci..

[91] Wang F., Man J.K.M., Lee E.-K.O., Wu T., Benson H., Fricchione G.L., Wang W., Yeung A. (2013). The Effects of Qigong on Anxiety, Depression, and Psychological Well-Being: A Systematic Review and Meta-Analysis. Evid. Based Complement. Altern. Med..

[92] Peters M.L. (2015). Emotional and cognitive influences on pain experience. Mod. Trends Psychiatry.

[93] Bresler D. (2005). Physiological Consequences of Guided Imagery. Pract. Pain Manag..

[94] Campbell K.L., Winters-Stone K.M., Wiskemann J., May A.M., Schwartz A.L., Courneya K.S., Zucker D.S., Matthews C.E., Ligibel J.A., Gerber L.H. (2019). Exercise Guidelines for Cancer Survivors: Consensus Statement from International Multidisciplinary Roundtable. Med. Sci. Sports Exerc..

[95] Mazzocco K., Milani A., Ciccarelli C., Marzorati C., Pravettoni G. (2023). Evidence for choosing Qigong as an Integrated Intervention in Cancer Care: An umbrella review. Cancers.

[96] Challinor J.M., Galassi A.L., Al-Ruzzieh M.A., Bigirimana J.B., Buswell L., So W.K.W., Steinberg A.B., Williams M. (2016). Nursing’s potential to address the growing cancer burden in low- and Middle-Income countries. J. Glob. Oncol..

